# Synthetic Applications of Oxidative Aromatic Coupling—From Biphenols to Nanographenes

**DOI:** 10.1002/anie.201904934

**Published:** 2019-12-03

**Authors:** Marek Grzybowski, Bartłomiej Sadowski, Holger Butenschön, Daniel T. Gryko

**Affiliations:** ^1^ Institute of Organic Chemistry Polish Academy of Sciences Kasprzaka 44/52 01-224 Warsaw Poland; ^2^ Institut für Organische Chemie Leibniz Universität Hannover Schneiderberg 1B 30167 Hannover Germany

**Keywords:** biaryls, Lewis acids, nanographenes, oxidative coupling, Scholl reaction

## Abstract

Oxidative aromatic coupling occupies a fundamental place in the modern chemistry of aromatic compounds. It is a method of choice for the assembly of large and bewildering architectures. Considerable effort was also devoted to applications of the Scholl reaction for the synthesis of chiral biphenols and natural products. The ability to form biaryl linkages without any prefunctionalization provides an efficient pathway to many complex structures. Although the chemistry of this process is only now becoming fully understood, this reaction continues to both fascinate and challenge researchers. This is especially true for heterocoupling, that is, oxidative aromatic coupling with the chemoselective formation of a C−C bond between two different arenes. Analysis of the progress achieved in this field since 2013 reveals that many groups have contributed by pushing the boundary of structural possibilities, expanding into surface‐assisted (cyclo)dehydrogenation, and developing new reagents.

## Graphical abstract

**Coupling up**: The dehydrogenative coupling of arenes is an ultimate example of C−H activation. This Review highlights the most exciting examples of the Scholl reaction, ranging from the synthesis of biphenols through natural products to nanographenes and on‐surface dehydrogenation reported since 2013.

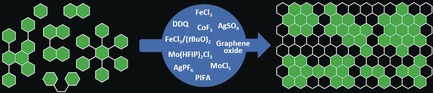

## 1 Introduction

**Scheme 1:**
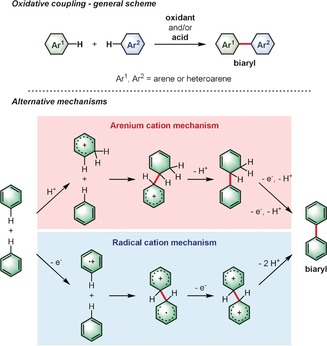
General scheme for the oxidative coupling of arenes through the two alternative, generally accepted, mechanisms using the formation of biphenyl as an example.

Oxidative aromatic coupling and the Scholl reaction, both discovered more than 100 years ago,^1^, ^2^, ^3^ have not lost any attraction to organic chemists in the last decade, despite the appearance of many modern C−H activation procedures. These reactions are often the methods of choice for the synthesis of large π‐extended scaffolds as well as smaller benzenoid or heteroaromatic compounds possessing biaryl linkages (Scheme 1). The Scholl reaction is undoubtedly the most useful for the construction of nanographenes^4^, ^5^ and their heterocyclic analogues.^6^ Indeed, in some cases, more than 100 C−C bonds are formed in one synthetic operation to furnish truly amazing polycyclic aromatic hydrocarbons from suitable precursors. There is no other reaction that can duplicate this result for a discrete molecule (i.e. not a polymer). However, despite ubiquitous utilization, the ability to foresee when and how this reaction will occur is still lacking. Indeed, the love‐hate relationship between synthetic organic chemists and dehydrogenative coupling is strongly related to the fact that it works with stunning efficiency in some cases, and that it fails in many others. Although we presented a comprehensive overview on the century‐long history of the Scholl reaction in 2013,^3^ a significant amount of experimental data in various versions of oxidative aromatic coupling has been accumulated within the last seven years. At the same time, these studies are increasingly accompanied by DFT calculations, which often, post‐factum, prove that the unanticipated course of a reaction really was to be expected as far as energies of transition states are concerned. Unfortunately, because of space limitations, not all the interesting results could be included in this Review. The photocyclodehydrogenation of stilbenes and derivatives of *o*‐terphenyls,^7^, ^8^ although very interesting and often complementary to the Scholl reaction, will not be described here for this reason. This Review is comprised of four sections that deal with intermolecular oxidative aromatic coupling, the intramolecular Scholl reaction, on‐surface (cyclo)dehydrogenation, and an outlook summarizing progress from 2013 until April 2019.

Scheme 1 depicts the general case of the oxidative coupling of arenes and explains the two generally accepted mechanisms: via arenium cation and radical cation intermediates.

## 2 Intermolecular Oxidative Aromatic Coupling

In the following section we will focus on key achievements in the field of intermolecular oxidative aromatic coupling that have appeared in the literature since our previous review was published in 2013. Occasional references to earlier work will be mentioned where appropriate.

### 2.1 Oxidative Homocoupling of Arenes and Heteroarenes

Among the numerous oxidants typically used for the construction of a new C−C bond in an oxidative manner, the most frequently applied is iron(III) chloride.^9^ New reagents are, however, continually proposed for homocoupling processes under oxidative conditions. Indeed, a graphene oxide (GO)/BF_3_⋅OEt_2_ system is able to promote oxidative C−H/C−H coupling between two electron‐rich arenes in a selective way.^10^ The utility of boron(III)‐based Lewis acids presumably relies on the activation of epoxy sites on a GO surface to form active radical species. EPR studies support a radical pathway for this transformation. An oxidative coupling process for simple 1‐ or 2‐substituted naphthalene derivatives can also proceed in the presence of AgSO_4_,^11^ albeit in moderate yields in most cases. Nevertheless, under these conditions, 1‐(trifluoromethyl)naphthalene gives 5,5′‐bis(trifluoromethyl)‐1,1′‐binaphthyl in 17 % yield, thus showing that the oxidizing power of silver(II)‐based systems can partially overcome the electronic limitations caused by an electron‐withdrawing CF_3_ group. The corresponding binaphthyls derived from 1‐nitro‐ and 1‐cyanonaphthalene are not formed under these conditions.

**Figure 1:**
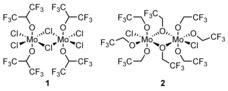
Structures of newly designed Mo‐based mediators for oxidative coupling.^16^

Molybdenum pentachloride is a selective, one‐electron oxidizing reagent frequently employed in many batch oxidative coupling processes,^12^ and more recently under continuous‐flow conditions.^13^ In most MoCl_5_‐mediated reactions of this type, undesired chlorination can be successfully avoided, as the C−C coupling is much faster than other side reactions. However, for highly electron‐rich substrates or when the coupling process proceeds slowly, chlorination becomes an issue.^14^ The application of MoCl_5_ together with a Lewis acid (usually TiCl_4_), which is believed to bind the coformed chloride anions, increases the overall efficiency of the C−C coupling,^15^ but this beneficial effect is not general. To solve this problem, the Waldvogel group designed^16^ the two dinuclear molybdenum(V) complexes **1** and **2** (Figure 1), which were prepared from MoCl_5_ and either 1,1,1,3,3,3‐hexafluoroisopropanol (HFIP) or 2,2,2‐trifluoroethanol (TFE) in 95 % and 97 % yield, respectively. According to electrochemical measurements and DFT calculations, only complex **1** has an oxidizing power comparable to that of MoCl_5_ [*E*_p_(MoCl_5_)=1.16 V; *E*_p_(**1**)=1.22 V, versus FcH/FcH^+^; FcH=ferrocene], while electron transfer from 3,4‐dimethoxytoluene to complex **2** should be strongly prevented because of the low potential value [*E*_p_(**2**)=0.31 V, versus FcH/FcH^+^] and the sterically congested coordination sphere. Problematic substrates were subjected to oxidative coupling reactions mediated by MoCl_5_, MoCl_5_/TiCl_4_, or complex **1** (Table 1). In all cases, the authors obtained considerably higher yields for reactions mediated by complex **1** compared with the other two systems and this fact was associated with such factors as the lower chlorine content of the reagent **1** (thus avoiding chlorination), the lower nucleophilicity of the HFIP ligands compared to chloride, and the considerably higher reaction rate of the C−C coupling compared with side processes induced by the higher oxidation potential of complex **1**.

**Table 1:** Comparison of the efficiencies of oxidative coupling reactions for different molybdenum(V)‐based systems.^16^

Compound		Yield [%]^[a]^		
		MoCl_5_^[c]^	MoCl_5_/TiCl_4_^[d]^	**1** ^[e]^
	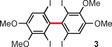	47	41	60
	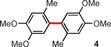	67	44	82
	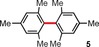	34	<5^[b]^	78

[a] Yields of isolated products. [b] Yield determined by GC. [c] 2.2 equiv of MoCl_5_. [d] 1.4–2.2 equiv of both MoCl_5_ and TiCl_4_. [e] 0.7–1.1 equiv of **1**.

It is not only molybdenum(V) complexes that are useful mediators in the oxidative formation of new C−C bonds. An electrochemical system consisting of an active molybdenum anode, a graphite cathode, and HFIP as a mediator was found to be highly effective in the dehydrogenative homocoupling of simple electron‐rich arenes.^17^ The elaborated method tolerates a broad scope of arenes and is particularly important, especially for environmental reasons, as it does not generate a large amount of reagent waste.

**Scheme 2:**
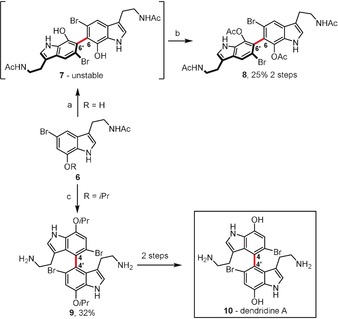
Reagents and conditions: a) (*t*BuO)_2_ (1.5 equiv), toluene, 130 °C; b) Ac_2_O, pyridine; c) MoCl_5_ (4 equiv), TiCl_4_ (4 equiv), 0 °C.

An interesting discovery was made by Boyd and Sperry,^18^ who examined the influence of various oxidizing agents on the dimerization process of hemidendridine acetates of type **6** (Scheme 2). Under the influence of a wide range of typical oxidants [FeCl_3_⋅SiO_2_, Ag_2_O, Pb(OAc)_4_, K_3_[Fe(CN)_6_], PhI(OAc)_2_ (PIDA)] **6** decomposes, and no trace of a dimer is detected in the reaction mixture. Nevertheless, the 6,6′‐coupled, symmetrical dimer **8** is formed exclusively through the oxidative dimerization of **6** mediated by (*t*BuO)_2_ in toluene at 130 °C followed by protection of the hydroxy groups in **7**. This is the result of an *ortho*‐*ortho* coupling, which is usually observed for phenols in the absence of steric hindrance^19^ or catalyst control.^20^ Completely different regioselectivity is observed if an indole precursor bears an *i*PrO substituent. Under Scholl‐type conditions, the best result was obtained with the MoCl_5_/TiCl_4_ system, thus **6** was successfully transformed into 4,4′‐bistryptamine **9** in 32 % yield. Besides **9**, several other unsymmetrical products were isolated from the reaction mixture in a total yield of 14 %. In this case, the reaction outcome is undoubtedly governed by the bulky O*i*Pr group, which enforces the dominant *para*–*para* coupling. Such a regioselectivity can also be achieved by blocking the *ortho* positions relative to the OH group, as was recently shown by Porco and co‐workers.^21^

**Figure 2:**
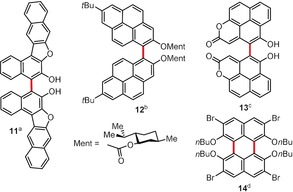
Reagents and conditions of oxidative dimerization: a) Cu(NO_3_)_2_⋅3 H_2_O (2 equiv), phenylethylamine, THF/MeOH, RT; b) FeCl_3_ (4 equiv), EtOH, reflux or Cu(BF_4_)_2_⋅*n* H_2_O (4 equiv), MeCN, reflux; c) FeCl_3_ (5 mol %), (*t*BuO)_2_ (2 equiv), DCE, 100 °C; d) 1. KMnO_4_ (0.25 equiv), MeOH/H_2_O, RT; 2. Na_2_S_2_O_4_, MeOH, RT; 3. BuBr, KOH, TBAB, dioxane, 80 °C. DCE=1,2‐dichloroethane, TBAB=tetrabutylammonium bromide.

The oxidative dimerization of larger phenols represents one of the most convenient pathways toward complex 2,2′‐dihydroxybiaryl compounds. According to the Tsubaki group, butterfly‐shaped molecule **11** can be obtained by the Cu^II^‐mediated dimerization of dinaphthofuran‐6‐ol (Figure 2).^22^ The dihedral angle between the two central naphthyl moieties can be altered by chemical modification of the central region; for example, acid‐catalyzed dehydration involving the central OH groups delivers an additional furan system, which in turn decreases the dihedral angle from 86.4° to 14.6°, as predicted by DFT methods. The protected 1,1′‐bi‐2‐pyrenol (**12**, Figure 2) can be prepared by the Fe^III^‐23 or Cu^II^‐mediated^24^ oxidative dimerization of the protected precursor of 2‐pyrenol. After dimerization, diastereomers of type **12** can be effectively separated^24^ using conventional chromatography and then be deprotected to deliver optically pure 1,1′‐bi‐2‐pyrenols. Another complex phenol **13**, based on the naphtho[2,1,8‐*def*]coumarin scaffold, was synthesized by employing a FeCl_3_/(*t*BuO)_2_ system.^25^ Numerous attempts to perform the second coupling process to generate a heterocyclic analogue of 2,4:9,11‐dinaphthoperylene were ineffective. In contrast, 3,6‐dibromo‐2,7‐dihydroxynaphthalene dimerizes selectively^26^ at C1 and C8 to form a mixture of dihydroxyperylenequinones, which after reduction and alkylation affords the octasubstituted perylene analogue **14**.

**Scheme 3:**
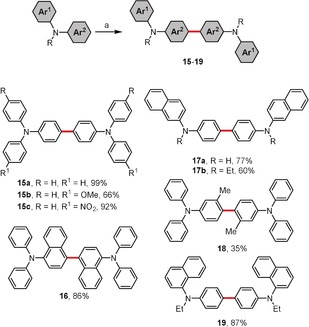
Reagents and conditions: a) *p*‐chloranil (2 equiv), MsOH/CH_2_Cl_2_, 0–25 °C. MsOH=methanesulfonic acid.

Anilines, as a consequence of their electron‐rich character and thus lower oxidation potentials, should undergo oxidative aromatic coupling more easily than phenolic derivatives. Indeed, treatment of some secondary and tertiary anilines with *p*‐chloranil as an oxidizing agent^27^ produces dimerization products **15**–**19** (Scheme 3). From a structural point of view, the method tolerates a variety of functional groups (even those with electron‐withdrawing character) as well as substrates with sterically hindered reactive sites. In parallel, Kita and co‐workers proved^28^ that the dimerization of 1‐naphthylarylamines, 1‐naphthyldiarylamines, or 2‐naphthyldiphenylamine mediated by phenyliodine(III) bis(trifluoroacetate) (PIFA) takes place selectively at the naphthyl site of the molecule. Similar to the *p*‐chloranil‐mediated method, these conditions are also applicable when steric hindrance is present within a substrate molecule.

**Scheme 4:**
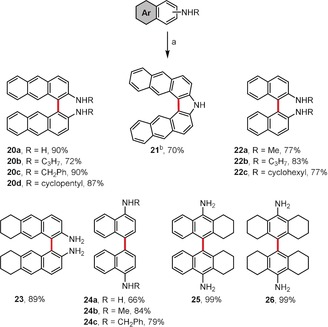
Reagents and conditions: a) Rh/C (5 mol %), TFA, air, RT; b) Rh/C (5 mol %), HFIP, air, RT. TFA=trifluoroacetic acid.

Scheme 4 depicts a catalytic approach for the oxidative homocoupling of anilines.^29^ In this case, a system comprised of rhodium adsorbed on carbon, air, and TFA as a solvent showed the highest efficiency. Biaryl amines **20**–**26** (including π‐expanded analogues **20 a**–**d**) were obtained selectively in high to excellent yields (up to 99 %). Interestingly, when HFIP was used as a solvent, the authors obtained the carbazole‐based compound **21** as the sole product in 70 % yield. The oxidative cross‐coupling of aromatic amines was also reported by Pappo and co‐workers as well as the Shindo group.^30^, ^31^

**Scheme 5:**
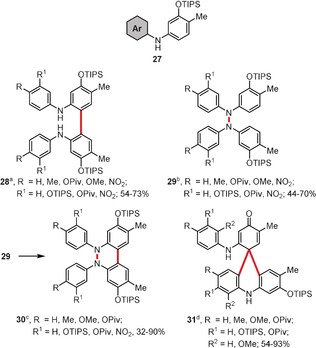
Reagents and conditions: a) [FeF_16_Pc] (1 mol %), MsOH, air, CH_2_Cl_2_, RT; b) [FeF_16_Pc] (5 mol %), NEt(*i*Pr)_2_, air (1 atm), DCE, 60 °C; c) [FeF_16_Pc] (10 mol %), AcOH, air, CH_2_Cl_2_, RT; d) FeCl_3_⋅6 H_2_O (2.4 equiv), CH_2_Cl_2_, reflux, argon. Piv=pivaloyl, TIPS=triisopropylsilyl.

In 2017, Knölker and co‐workers investigated the reactivity of diarylamines of type **27** towards iron(III)‐based catalysts: hexadecafluorinated iron‐phthalocyanine (FeF_16_Pc) and iron(III) chloride hexahydrate (Scheme 5).^32^ By using FeF_16_Pc as a catalyst, 2,2′‐di(arylamino)biaryls **28**, tetraarylhydrazines **29**, and 5,6‐dihydrobenzo[*c*]cinnolines **30** can be obtained in reasonable yields. The selectivity is governed by the use of acidic or basic additives. Namely, in the presence of a FeF_16_Pc/MsOH system, diarylamines **27** give exclusively the 2,2′‐coupled products **28**. By changing the character of the additive from acidic to basic, **27** undergoes N−H/N−H oxidative coupling to form the tetraarylhydrazines **29**. Unexpectedly, when **27** was treated with a stoichiometric amount of FeCl_3_⋅6 H_2_O, spiro‐products **31** were obtained in reasonable yields.

Recently, Venkatakrishnan and co‐workers^33^ found that, whereas carbazoles containing electron‐donating groups form 3,3′‐coupled products, substrates bearing electron‐withdrawing groups tend to form predominantly 1,1′‐coupled molecules. The dimerization of *N*‐phenylcarbazole can also be achieved using Eaton's reagent (composed of 7.5 wt % phosphorus pentoxide in methanesulfonic acid).^34^

The synthesis of large graphenic structures from relatively simple molecules is usually not a straightforward task. Among the many approaches leading to nanographenes, the benzene ring seems to be an ideal candidate for the bottom‐up synthesis of such structures.^5^, ^6^, ^35^, ^36^ Zarabin and co‐workers attempted^37^ the construction of nanographene sheets from benzene at a liquid–liquid interface. According to their strategy, a single benzene ring should undergo FeCl_3_‐mediated oxidative coupling to give oligo(paraphenylene) and/or poly(paraphenylene). The poly(paraphenylene) fractions should spontaneously migrate to the water–benzene interface, where the second polymerization step that creates lateral chains occurs. In the final step, a series of intramolecular oxidative coupling processes between adjacent phenyl rings within the branched polymer takes place. The proposed method allows the preparation of large graphene nanosheets with a surface area of about 800 nm^2^.

Recently, oxidized active carbon/oxygen,^38^ 2,3‐dichloro‐5,6‐dicyano‐1,4‐benzoquinone (DDQ)/MsOH,^39^ and NOBF_4_/oxygen^40^ systems were found to be applicable for the construction of 2,2′‐ or 3,3′‐coupled motifs starting from benzofused heterocycles such as indole, benzothiophene, or benzofuran.

**Scheme 6:**
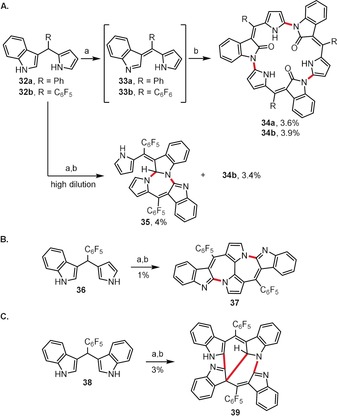
Reagents and conditions: a) DDQ (2.2 equiv), THF, RT; b) Cu(OAc)_2_ (1 equiv), THF, RT.

Short‐chain oligopyrroles constitute outstanding substrates for the formation of various pyrrolic macrocycles, which may vary significantly in their electronic properties and metal‐binding properties.^41^, ^42^, ^43^ Anand and co‐workers investigated the reactivity of benzo‐dipyrromethanes under oxidative conditions (Scheme 6),^44^ and they uncovered that the reaction outcome highly depends on the exact structure of the dipyrromethene substrate. The DDQ‐mediated oxidation of monobenzo‐dipyrromethanes **32** to the corresponding dipyrromethenes **33** (Scheme 6 A) followed by treatment with Cu(OAc)_2_ leads to cyclic trimers of type **34**. If the same sequence was applied to **32 b** in high dilution, a mixture of dimer **35** and trimer **34 b** could be obtained. Subjecting **36** to the same reaction sequence (Scheme 6 B) resulted in cyclodimer **37** containing two fused seven‐membered rings in the central part of the molecule. Finally, the stepwise, DDQ/Cu(OAc)_2_‐mediated oxidation of dibenzo‐dipyrromethane **38** gives access to the unsymmetrical, acyclic dimer **39** (Scheme 6 C) with six‐ and seven‐membered rings at its core.

**Scheme 7:**
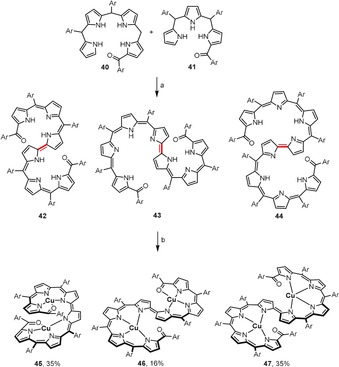
Reagents and conditions: a) DDQ (4 equiv), CH_2_Cl_2_, RT; b) Cu(OAc)_2_, CH_2_Cl_2_/MeOH, RT. Yields after two steps. Ar=pentafluorophenyl.

Oxidative homo‐ and cross‐coupling processes are key synthetic steps towards helical π‐conjugated oligopyrrins with a bipyrrole linkage (Scheme 7).^45^ A series of DDQ‐mediated oxidative couplings between the monoacylated bilane **40** and/or tripyrrane (**41**) lead to oligopyrrolic molecules **42**–**44** containing six, seven, or eight pyrrolic units, respectively. These oligopyrrolic derivatives readily undergo double complexation with copper(II) acetate to form bis(copper(II)) complexes, which distinctly differ in geometry. Complex **45** adopts a helical coil geometry, whereas complexes **46** and **47** exhibit S‐shaped open‐spiral‐type arrangements.

### 2.2 Oxidative Cross‐Coupling of Arenes and Heteroarenes

**Scheme 8:**
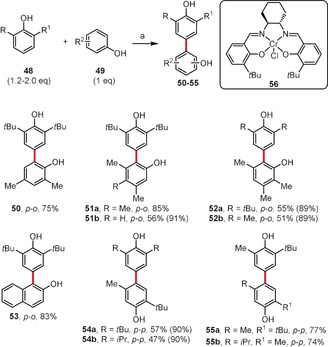
Reagents and conditions: a) **56** (5 mol %), O_2_, DCE, 50–85 °C. Yields in parentheses are based on recovered substrate.

Cross‐coupling processes occurring between two different arenes could potentially afford three different products (or more, if a given substrate has two inequivalent reactive sites). To reduce the number of possible products, Pappo's model^46^ based on an interplay between global nucleophilicity and oxidation potentials may be considered. Another system consisting of a salen‐based catalyst (**56**) and atom‐economical oxygen as the terminal oxidant was found to be broadly effective in oxidative cross‐coupling reactions to give compounds **50**–**55** (Scheme 8).^20^ According to Kozlowski, the 2,6‐disubstituted phenol **48** should be applied as the substrate to carry out a selective cross‐coupling process and reduce the number of possible products.^47^ It was assumed that such a phenol (called Type I) should bind selectively at its *para* site to a less‐hindered site of a metal‐bound radical or radical cation of the second phenolic partner (**49**). Taking into account the regioselectivity of the method, substrates of type **49** were assigned as Type II (2,4‐, 3,4,5‐, 3,5‐, and 2,3,5‐substituted phenols) and Type III (2,5‐substituted phenols), as they provide *para*–*ortho* (*p‐o*) and *para*–*para* (*p‐p*) coupled products, respectively.

Electrochemical oxidation is currently being considered as a green alternative for classical oxidative aromatic coupling, because such a method does not require a metal‐based catalyst or a stoichiometric amount of an oxidant to form the crucial intermediate—a radical cation. However, the selective generation of a new C−C bond between two different aryl moieties under either classical or electrochemical conditions is challenging due to a strong tendency to form homocoupled products.

Recently, Waldvogel and co‐workers proved that the use of HFIP as a mediator, combined with a boron‐doped diamond electrode (BDD) as the anode constitutes a powerful system for selective phenol–arene cross‐coupling under electrochemical conditions.^48^, ^49^, ^50^, ^51^, ^52^ The importance of this specific solvent and the source of selectivity can be attributed to the strongly different solvation of the individual coupling partners and, therefore, the decoupling of the oxidation potential from the nucleophilicity. The efficiency and selectivity of the cross‐coupling process can be improved when HFIP/methanol or HFIP/water mixtures are used as mediators.^53^, ^54^ For example, the electrochemical oxidation of N‐protected aniline derivatives using HFIP/methanol as a mediator and glassy carbon as the anode material leads to a variety of unsymmetrical 2,2′‐diaminobiaryls in moderate to good yields and excellent regioselectivity.^55^ Easily removable protecting groups including Boc, acetyl, benzoyl, pivaloyl, and trifluoroacetyl are fully compatible with the electrochemical environment; thus, after deprotection under mild conditions, this method provides expedient access to important structural motifs.

Recently, Waldvogel and co‐workers successfully elaborated an electrochemical alternative for the arylation of sulfur‐containing heterocycles. Thiophenes^56^ and benzothiophenes^57^ were effectively coupled with phenol derivatives to form biaryl systems with high selectivity and efficiency. Importantly, with 2‐ as well as 3‐substituted benzothiophenes they obtained a broad scope of products in yields up to 88 %. In the case of thiophenes, applying an excess of phenolic substrate (3 equiv) leads to diarylated products in high yields.

**Figure 3:**
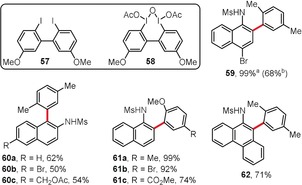
Reagents and conditions: a) **57** (5 mol %), mCPBA (1.5 equiv), TFE/CH_2_Cl_2_, RT; b) **58** (0.75 equiv), TFE/CH_2_Cl_2_, RT. mCPBA=*meta*‐chloroperbenzoic acid, TFE=2,2,2‐trifluoroethanol.

Hypervalent iodine(III)‐based compounds serve as excellent oxidizing agents in oxidative aromatic coupling processes,^58^, ^59^ and they are typically used in stoichiometric or excess amounts. The first organocatalytic version of an oxidative cross‐coupling reaction between sulfonylanilides and aromatic hydrocarbons was developed by the Kita group (Figure 3).^60^ During this process, the organocatalyst **57** based on a 2,2′‐diiodobiaryl scaffold is oxidized to its hypervalent iodine(III) analogue **58** by mCPBA. After the cross‐coupling reaction, the organocatalyst is then regenerated by the same means as its activation. The process is slower and a lower yield was obtained when 0.75 equiv of **58** was used instead of a catalytic amount of **57**. A series of derivatives **59**–**62** containing naphthyl or phenanthryl substituents were obtained using the organocatalytic method, with yields of up to 99 %.

In their follow‐up publication, the Kita group explored simple iodobenzenes as potential organocatalysts in oxidative phenol–arene or phenol–phenol cross‐coupling reactions.^61^ A catalytic system consisting of 4‐iodoanisole (10 mol %), oxone (1.2 equiv), 18‐crown‐6, and CH_3_COOH in HFIP was shown to be the most efficient method. High regioselectivity and broad substrate scope represent indisputable advantages of this method. According to the studies of More and Jeganmohan,^62^ oxone mixed with the phase‐transfer catalyst Bu_4_N^+^ HSO_3_^−^ in acetic acid also constitutes a selective system for the formation of unsymmetrical biphenols; however, the yields obtained were only moderate.

**Scheme 9:**
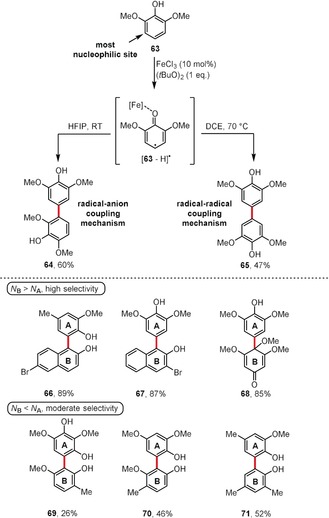
Pappo's model for selective oxidative cross‐coupling.^63^

Recently, Pappo and co‐workers made a significant contribution to this field by the discovery^46^, ^63^ that, depending on the solvent used during an oxidative coupling process, two different products (**64** and **65**) can be obtained starting from 2,6‐dimethoxyphenol (**63**; Scheme 9, top). In a HFIP solution, the initially formed radical [**63**−H]^.^ is stabilized to a greater extent than in DCE, thus it reacts with the most nucleophilic site of phenol **63** and leads to **64** through a radical–anion coupling mechanism. In DCE, [**63**−H]^.^ is poorly stabilized, and it undergoes a rapid recombination process through a radical–radical coupling mechanism. Based on this finding, the authors developed a predictive model based on the interplay between the theoretical global nucleophilicity (*N*) and the susceptibility to oxidation (derived from a direct comparison of the oxidation potentials measured in HFIP).^46^ According to this model, an efficient cross‐coupling process between phenols **A** and **B** can be performed as long as:

1. one of the phenols (**A**) can be selectively oxidized to a corresponding phenoxyl radical (*E*_ox_^**A**^ < *E*_ox_^**B**^) in the presence of the second phenolic partner **B**;
2. the second phenolic partner (**B**) exhibits a more pronounced nucleophilic character (that is when *N*_**B**_>*N*_**A**_ or Δ*N*>0).

A set of unsymmetrically coupled biaryls **66**–**68** was selectively constructed using this model when Δ*N*>0 (Scheme 9, bottom). When Δ*N*<0, the selectivity of this process decreases, thus only moderate yields are achieved for cross‐coupling reactions (e.g. products **69**–**71**). During the carbon–carbon bond‐forming step in the FeCl_3_/(*t*BuO)_2_/HFIP‐mediated process, both phenolic components are attached to the metal center. As an extension of the proposed model, the same group later discovered^64^ that a system consisting of an iron *meso*‐tetraphenylporphyrin chloride (Fe[TPP]Cl) complex and HFIP allows for the selective oxidative cross‐coupling of readily oxidized phenols with poorly nucleophilic phenolic partners (Δ*N*<0). It is believed that the high selectivity of the process results from the presence of a single available coordination site for phenol binding on the metal center in Fe[TPP]Cl.

**Figure 4:**
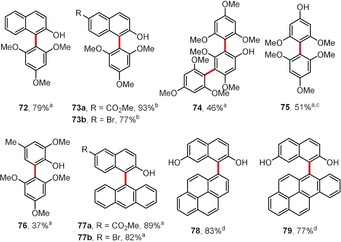
Reagents and conditions: a) FeCl_3_ (5–15 mol %), (*t*BuO)_2_ (1.5–2 equiv), HFIP, RT; b) FeCl_3_⋅6 H_2_O (5 mol %), (*t*BuO)_2_ (2 equiv), HFIP, RT; c) TFE was used as a solvent; d) 1‐phenyl‐2,2,2‐trifluoroethanol was used as a solvent at 40 °C.

The authors have demonstrated that the use of the iron(III)/(*t*BuO)_2_ couple in fluorinated solvents enables many valuable cross‐coupling products to be obtained, such as biaryls **72**–**73** and **75**–**76**, as well as triaryl **74** (46 % yield; Figure 4).^46^, ^63^ Importantly, polycyclic aromatic hydrocarbons larger than naphthalene, such as anthracene, pyrene, and benzopyrene, are also reactive in this transformation and lead to **77**–**79**. This study was recently further extended to include cross‐coupling catalyzed by Co^II^‐salen and Cr^III^‐salen complexes.^65^, ^66^

**Scheme 10:**
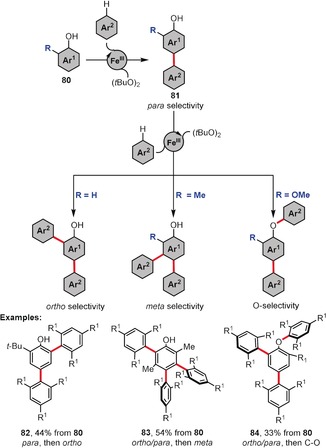
Catalyst and oxidant loadings: FeCl_3_ (15 mol %), (*t*BuO)_2_ (3–5 equiv). R^1^=OMe.

As shown in Figure 4, the reaction of 3,5‐dimethoxyphenol and 1,3,5‐trimethoxybenzene (2.1 equiv) gives the bisarylated product **74** in reasonable yield. Intrigued by this result, the same authors evaluated the dilemma of polyarylation in depth (Scheme 10).^67^ According to their study, the first coupling reaction of the phenolic substrate **80** occurs at the available *ortho*/*para* position(s) relative to the hydroxy group. Then, they found that the subsequent reaction strongly depends on the character of the substituent R. If R=H, the next coupling process leads to *ortho*‐arylated derivative **82**. In the case of R being a methyl group, the second/third arylation reaction exhibits *meta* selectivity, finally providing a compound with an exemplary substitution pattern of type **83**. When R is a methoxy group, the third arylation reaction tends to proceed chemoselectively at the OH group to form a C−O coupling product **84**. The EPR studies performed on the key persistent phenoxyl radicals clearly demonstrated that the coupling selectivity strongly correlates with the distribution of high spin density over the above‐mentioned radicals.

**Scheme 11:**
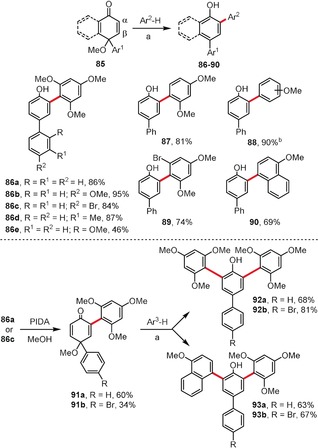
Reagents and conditions: a) FeCl_3_ (10 mol %), MeCN, RT; b) a mixture of regioisomers (*ortho*/*para*=1:2) was obtained.

Polyarylated phenolic derivatives can also be assembled by the iron(III)‐catalyzed functionalization of 4‐aryl‐4‐methoxy‐2,5‐cyclohexadienones **85** (Scheme 11, top).^68^ Although this reaction is not an oxidative aromatic coupling, but rather a polar reaction of an electrophile **85** with arene nucleophiles, we decided to present it here for the sake of completeness of strategies leading to polyarylated phenols. Substrates of type **85** were prepared using the PIDA‐mediated oxidation of the corresponding 4‐arylphenols in the presence of MeOH.^69^, ^70^ Initially, compounds **85** are arylated at the α‐position relative to the keto group in the presence of a catalytic amount of iron(III) chloride, thereby leading to the 2,4‐diarylphenolic derivatives **86**–**90**. However, for a substrate formed by the PIDA‐mediated oxidation of 4‐methyl‐1‐hydroxynaphthalene in MeOH they obtained a mixture of α‐ and β‐arylated products in yields of 76 and 20 %, respectively. Products of monoarylation, that is **86 a** or **86 c**, can be subsequently oxidized to **91 a**,**b** and arylated again to give 2,4,6‐triarylphenolic molecules **92**–**93** (Scheme 11, bottom). One major drawback of the above method is that a significant excess of the arene (Ar^2^H up to 10 equiv) is required to achieve monoarylation.

**Scheme 12:**
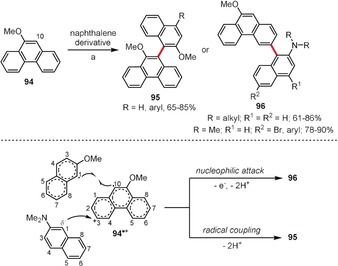
Reagents and conditions: a) (NH_4_)_2_S_2_O_8_ (6 equiv), TFA/CH_2_Cl_2_, 25 °C.

It is well known that 9‐phenanthrenol undergoes an oxidative aromatic homocoupling reaction at C10 under the influence of Cu^II^ species.^71^, ^72^ Wang et al. found^73^ that 9‐methoxyphenanthrene (**94**) shows a similar reactivity towards 2‐methoxynaphthalene derivatives in the presence of (NH_4_)_2_S_2_O_8_, with C10/C1‐linked molecules **95** being formed with excellent regioselectivity (Scheme 12). Interestingly, when 2‐naphthylamine derivatives were employed in this cross‐coupling reaction, C3‐arylation products **96** were obtained in good to excellent yields. According to DFT calculations employed in this study, **94** and 2‐methoxynaphthalene cation radicals have the highest spin density distribution at positions C10 and C1, respectively, and they follow a radical coupling pathway leading to the C10/C1 linked product (Scheme 12, bottom). In contrast, **94** reacts with 2‐naphthylamine at the most electrophilic position (C3), because the oxidation potential of naphthylamine increases significantly upon protonation in acidic media, thereby preventing the effective formation of a radical cation.

**Figure 5:**
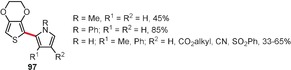
Cross‐coupling conditions: PIFA (1 equiv), Me_3_SiBr (2 equiv), HFIP, RT.

Kita and co‐workers successfully performed a series of PIFA‐mediated intermolecular oxidative coupling processes between pyrrole derivatives and 3,4‐ethylenedioxythiophene (Figure 5).^74^ The dimers of type **97** were synthesized in moderate to good yields with excellent regioselectivity. In the case of dimers containing an unprotected pyrrole unit, intramolecular hydrogen bonds enforce high coplanarity of both subunits, as proven by X‐ray crystallography, potentially allowing these dimers to serve as excellent substrates for efficient conducting polymers.

### 2.3 Enantioselective Synthesis of Chiral Biaryls

**Scheme 13:**
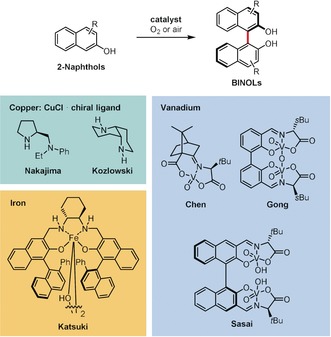
Early catalytic systems for the enantioselective synthesis of BINOL derivatives by the oxidative dimerization of 2‐naphthols.

Hindered rotation around the C_aryl_–C_aryl_ bond, such as in binaphthyl or *ortho*‐substituted biphenyl derivatives, gives rise to axial chirality, which is present in many natural products^75^, ^76^, ^77^, ^78^ and is the basis of various asymmetric catalytic systems.^79^ Hence, it is not surprising that the oxidative coupling of arenes, being one of the simplest ways to synthesize biaryls, was quickly recognized as a potential method to access optically active products.^47^, ^76^, ^78^ Early successful reports were based on the use of copper complexes with chiral amines as catalysts for the enantioselective synthesis of BINOL derivatives under aerobic conditions (Scheme 13).^80^, ^81^, ^82^, ^83^ The enantioselectivity was significantly improved after mononuclear^84^, ^85^ and binuclear^86^, ^87^ vanadium complexes were introduced.^88^ Recently, Egami and Katsuki developed chiral iron(salan) complexes (Scheme 13), which afforded unsubstituted BINOL and 3,3′‐disubstitued derivatives with an enantiomeric excess of 64 % and up to 97 %, respectively.^89^ Progress in the asymmetric synthesis of biaryls has been briefly summarized in recent articles.^47^, ^88^, ^90^, ^91^ The most important recent contributions are presented below.

**Scheme 14:**
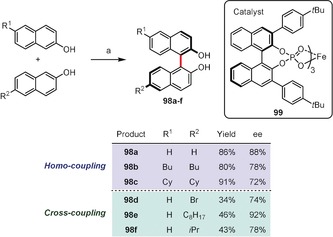
Reaction conditions: a) **99** (2.5 mol %), (*t*BuO)_2_ (1 equiv), DCE/HFIP 1:1, RT.

Pappo and co‐workers developed a new chiral iron complex **99** bearing three BINOL‐phosphate ligands which turned out to be a very efficient catalyst for the enantioselective homo‐ and cross‐coupling of 2‐naphthols.^92^ Scheme 14 presents selected examples of BINOLs (**98 a**–**f**) prepared with good *ee* values using as little as 2.5 mol % of catalyst **99** in a mixture of DCE and HFIP as a solvent and with di‐*tert*‐butyl peroxide (1 equiv) as a terminal oxidant. Importantly, the authors have also found that enantiopure BINOLs undergo racemization in the presence of Fe^III^ and Cu^II^ salts, which may reduce the enantiomeric excess of the products from the oxidative coupling. In the case of cross‐coupling, the reaction yields were significantly lower due to the formation of homocoupled side products. Nevertheless, this is one of only a few examples of enantioselective oxidative cross‐coupling reactions to date.

**Scheme 15:**
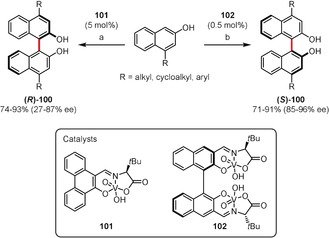
Reaction conditions: a) **101** (5 mol %), O_2_, CCl_4_, RT; b) **102** (0.5 mol %), O_2_, CH_2_Cl_2_, RT.

Dinuclear vanadium catalysts are among the most efficient examples for the enantioselective syntheses of BINOLs,^86^, ^87^, ^88^, ^93^ useful even in the dimerization of PAH‐based phenols.^94^ However, recent studies have revealed that chiral mononuclear vanadium(V) catalysts are also useful. Takizawa, Oh, and co‐workers found that 4‐substituted 2‐naphthols undergo oxidation with oxygen in the presence of mononuclear catalyst **101** or dinuclear **102** to give either *R* or *S* enantiomers, respectively, of BINOLs **100** in good yields and high *ee* values (Scheme 15).^95^ The mononuclear catalyst **101** had to be used in higher loadings (5 mol %) and resulted in slightly lower *ee* values.

**Scheme 16:**
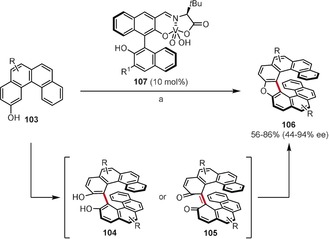
Reaction conditions: a) O_2_, CCl_4_, 50 °C. R=H, alkyl, aryl, Br; R^1^=3,5‐Ph_2_C_6_H_3_.

Compound **107**, also a mononuclear vanadium complex, was found by Takizawa, Sasai, and co‐workers to catalyze the enantioselective formation of oxa[9]helicenes **106** in one step from 2‐hydroxybenzo[*c*]phenanthrenes **103** (Scheme 16).^96^ In this transformation, **107** acts simultaneously as both a redox catalyst (enantioselective aerobic oxidative coupling) and Lewis acid (metal‐mediated intramolecular cyclization) catalyst. The authors proposed that this process may proceed via intermediates **104** or **105**, although these could not be detected in the reaction mixture. This method tolerates a broad scope of substrates, giving (*M*)‐oxa[9]helicenes with ee values up to 94 %.

**Scheme 17:**
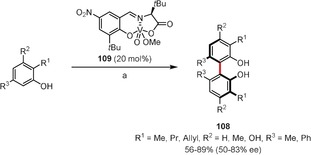
Reaction conditions: a) **109** (20 mol %), AcOH (6.25 equiv) or LiCl, O_2_, toluene, 0 °C or 25 °C.

Although numerous chiral catalysts have been developed for the asymmetric synthesis of BINOLs by oxidative coupling, the enantioselective preparation of chiral biphenyl derivatives has remained challenging until very recently. Kozlowski and co‐workers addressed this issue and performed an extensive catalyst optimization study using dinuclear vanadium catalysts as a starting point.^91^, ^97^ It turned out that nitro‐substituted mononuclear vanadium complex **109** actually provided the best results, especially in the presence of acetic acid or lithium chloride as additives (Scheme 17). A series of biphenols of type **108** were obtained in good yields and moderate *ee* values. *N*‐Benzyl‐2‐hydroxycarbazoles also reacted under similar conditions to give the corresponding 1,1′‐coupled dimers enantioselectively. Kozlowski and co‐workers utilized the new catalyst in the total synthesis of chaetoglobin, where a key chiral biaryl intermediate was prepared by atroposelective oxidative dimerization of phenols catalyzed by **109**.^98^

Very recently, Takizawa, Sasai, and co‐workers developed a dinuclear vanadium complex that mediated the oxidative homocoupling of various 5‐arylresorcinols to furnish the corresponding biaryls with up to 98 % *ee*.^99^

### 2.4 Metal‐Catalyzed and Metal‐Mediated Intermolecular Oxidative Coupling of Electron‐Poor Arenes

In terms of both the mechanism and the scope, the metal‐catalyzed intermolecular dehydrogenative coupling of electron‐poor arenes differs significantly from classic oxidative aromatic coupling. However, we have decided to include an overview of this synthetic methodology, with an emphasis on copper and rhodium catalysis as well as a few selected examples of palladium‐mediated reactions which have been more generally reviewed recently.^100^, ^101^, ^102^, ^103^, ^104^

#### 2.4.1 Copper

**Figure 6:**
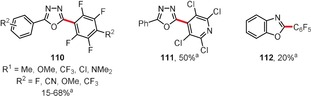
Reaction conditions: a) Heterocycle (1 equiv), halogenated arene (5–30 equiv), CuBr (1 equiv), 1,10‐phenantroline (1 equiv), *t*BuOLi, O_2_, CH_3_CN, RT.

In 2013, Bolm and co‐workers reported interesting copper‐mediated oxidative coupling reactions of 1,3,4‐oxadiazoles and benzoxazoles with pentafluorobenzene and its analogues (Figure 6).^105^ Compounds **110**–**112** were obtained in yields of 15–68 %.

**Scheme 18:**
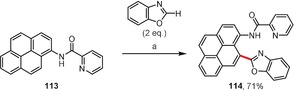
Reaction conditions: a) Cu(OAc)_2_ (3 equiv), PivOH (1 equiv), mesitylene, 165 °C.

Miura and co‐workers realized the copper‐mediated regioselective dehydrogenative biaryl coupling of naphthylamides and 1,3‐azoles using copper(II) acetate in the presence of pivalic acid.^106^ As a representative example, the reaction of **113** with benzoxazole leading to **114** is given in Scheme 18. Deuterium labeling experiments suggest that the metalation takes place at the benzoxazole first, with the species formed then undergoing a directed C−H activation at the naphthalene derivative.

#### 2.4.2 Rhodium

**Figure 7:**
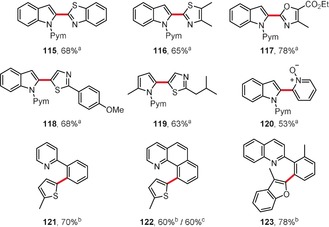
Reaction conditions: a) indole (1 equiv), heteroarene (3 equiv), [Cp*RhCl_2_]_2_ (2.5 mol %), AgSbF_6_ (10 mol %), Ag_2_CO_3_ (2 equiv), PivOH (2 equiv), DMF, 150 °C; b) pyridine derivative (1 equiv), heteroarene (3 equiv), [Cp*RhCl_2_]_2_ (5 mol %), AgSbF_6_ (20 mol %), Cu(OAc)_2_ (3 equiv), DCE, 140 °C; c) pyridine derivative (1 equiv), heteroarene (3 equiv), [Ru(*p*‐cymene)Cl_2_]_2_ (5 mol %), KPF_6_ (20 mol %), Cu(OAc)_2_⋅H_2_O (2 equiv), DCE, 140 °C. Cp*=C_5_Me_5_, PivOH=pivalic acid, Pym=2‐pyrimidyl.

In the last few years some interesting reports on rhodium‐catalyzed dehydrogenative arene coupling reactions have appeared, often within the context of directed C−H activation. You, Lan, and co‐workers reported the chelation‐assisted rhodium(III)‐catalyzed oxidative C−H/C−H cross‐coupling of indoles and pyrroles with heteroarenes preferentially at the C2‐position (Figure 7).^107^ Their strategy allows the selective coupling between an electron‐rich heteroarene with a directing group and both electron‐rich and electron‐deficient heteroarenes. For example, 2‐pyrimidyl‐protected indoles or 2‐methylpyrrole react with electron‐poor heterocycles in the presence of a [Cp*RhCl_2_]_2_/AgSbF_6_ catalyst system to afford coupling products **115**–**120**. Closely related reactions were published by Kambe and co‐workers at almost the same time.^108^ More recently, the procedure has been extended to the synthesis of mono‐ and bisarylated phenols.^109^

In the context of directed C−H activation, You and co‐workers also reported the rhodium‐ or ruthenium‐catalyzed oxidative C−H/C−H cross‐coupling between various heterocycles and 2‐aryl‐substituted pyridines or quinolines in the presence of [Cp*RhCl_2_]_2_ or [{Ru(*p*‐cymene)Cl_2_}_2_] as catalysts.^110^ Three exemplary products **121**–**123** are presented in Figure 7. Similar reactions were reported with oxime ethers as substrates.^111^ Later the same group reported the use of the Wilkinson catalyst for the *ortho*‐heteroarylation of pivaloyl anilides.^112^

**Scheme 19:**
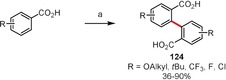
Reaction conditions: a) [Rh(nbd)Cl]_2_ (5 mol %), MnO_2_ (3 equiv), H_2_O, air, 150 °C.

Li and co‐workers developed rhodium(I) catalysts for the regiospecific homodehydrogenative coupling of aromatic carboxylic acids in water (Scheme 19).^113^, ^114^ The dimerization of variously substituted benzoic acids upon reaction with the chloro(norbornadiene)rhodium(I) dimer as a catalyst and manganese dioxide or sodium chlorite as the oxidant gave coupling products of type **124** in good yields. In a subsequent report, the authors showed that cross‐dehydrogenative coupling is also possible under similar reaction conditions if one of the carboxylic acids is electron‐rich.^115^

#### 2.4.3 Palladium

**Figure 8:**
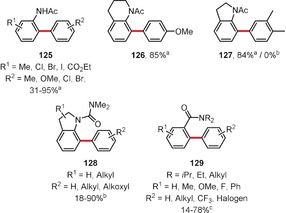
Reaction conditions: a) anilide (1 equiv), arene (20 equiv), Pd(OAc)_2_ (10 mol %), (NH_4_)_2_S_2_O_8_ (2 equiv), TFA (20 equiv), RT; b) Pd(OAc)_2_ (10 mol %), Cu(OAc)_2_ (1 equiv) or O_2_, TFA, arene as solvent, 50 °C; c) PdCl_2_ (10 mol %), AgOTf (20 mol %), NaOTf (20 mol %), K_2_S_2_O_8_ (2 equiv), DMA (2 equiv), arene as solvent, 80 °C. DMA=*N*,*N*‐dimethylacetamide.

Most of the reported metal‐catalyzed or ‐mediated C−H/C−H dehydrogenative coupling reactions involve palladium as the catalyst metal. There are some reports on palladium‐catalyzed arylations by coupling an arene with another bearing an *ortho* directing group.^100^, ^101^, ^102^, ^103^, ^104^ In this context, Song, You, and co‐workers reported reactions of anilide derivatives with arenes, which were catalyzed by palladium(II) acetate in the presence of ammonium peroxodisulfate as the oxidant and TFA as the solvent (Figure 8).^116^ Cross‐coupled products such as **125**–**127** were obtained in yields ranging from 31 % to 95 %. A closely related reaction, namely the palladium(II)‐catalyzed dehydrogenative coupling of arenes with indolines at C7, has also been reported (products **128**; Figure 8).^117^

Guan and co‐workers showed that biaryls of type **129** can be synthesized by coupling tertiary benzamides with arenes by utilizing the in situ generated palladium(II) triflate as the catalyst and dipotassium peroxodisulfate as the oxidant (Figure 8).^118^

**Figure 9:**
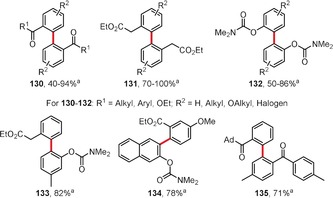
Reaction conditions: a) Pd(OAc)_2_ (7.5 mol %), NaIO_4_ (0.6 equiv), K_2_S_2_O_8_ (0.5 equiv), TfOH, HFIP, 25–70 °C. TfOH=trifluoromethanesulfonic acid.

Homo‐ and cross‐dehydrogenative coupling reactions yielding biaryls were also reported by Zhang and Rao, who used HFIP as the solvent and sodium periodate/dipotassium peroxodisulfate as oxidants.^119^ Homodimers of types **130**–**132** as well as unsymmetric biaryls (e.g. **133**–**135**) were obtained in good yields (Figure 9).

In an interesting mechanochemical approach, Xu and co‐workers showed that the rates of dehydrogenative coupling reactions can significantly increase when ball milling is used instead of conventional heating. Long reaction times of up to 24 h for conventional reactions were reduced to 1 h, without the need for external heating.^120^

**Scheme 20:**
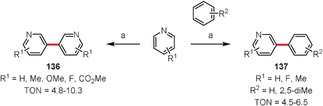
Reaction conditions: a) Pd(OAc)_2_ (10 mol %), 1,10‐phenanthroline (10 mol %), Ag_2_CO_3_ (2 equiv), K_2_CO_3_ (2 equiv), pyridine, 130 °C.

Although the dehydrogenative coupling reactions mentioned so far involve benzene derivatives that result in biphenyl‐derived products, there are also reports on the coupling of heterocycles. Yu and co‐workers reported an interesting coupling reaction of pyridines to afford 3,3′‐bipyridyl derivatives (e.g. **136**) in addition to a minor amount of the 2,3′‐isomer.^121^ The reaction is catalyzed by palladium(II) acetate in the presence of 1,10‐phenanthroline and gives the homocoupling products with turnover numbers (TONs) of up to 10.3 (Scheme 20). Cross‐coupling reactions of pyridines with benzene derivatives to give 2‐phenylpyridines **137** were also reported.

## 3 Intramolecular Oxidative Aromatic Coupling

Although numerous impressive examples have been known for a long time, only in recent years has the methodology for the intramolecular oxidative coupling of arenes reached its synthetic potential and become a well‐established tool used in a variety of applications. The growing importance of research in such fields as organic electronics, nanotechnology, and bioimaging, has led to an increased demand for simple and effective methods for the creation of intramolecular C_aryl_–C_aryl_ bonds. Often simply (but not entirely correctly) referred to as the “Scholl reaction”, the intramolecular oxidative coupling of arenes is a versatile and widely used method for the synthesis of various polycyclic aromatic hydrocarbons (PAHs), including so‐called nanographenes, as well as expansions/planarizations of π‐conjugated systems and for the preparation of strained, curved, or twisted systems. For space reasons, the application of cyclodehydrogenation in the synthesis of small‐molecule targets is presented in the Supporting Information.

### 3.1 Large Planar PAHs, Expanded Acenes, and Nanographenes

Perhaps there is no better way to demonstrate the potential of intramolecular oxidative coupling methods than in the syntheses of large polycyclic aromatic hydrocarbons and nanographenes, where multiple (sometimes hundreds or even thousands) C_aryl_–C_aryl_ bonds are formed in a single operation. It is easy to imagine that a similar goal would be impossible to achieve using alternative methods of C_aryl_–C_aryl_ coupling, since it would require the presence of numerous activating units (e.g. halides, boronic acids/esters, etc.) strategically positioned over the precursor molecules.

**Figure 10:**
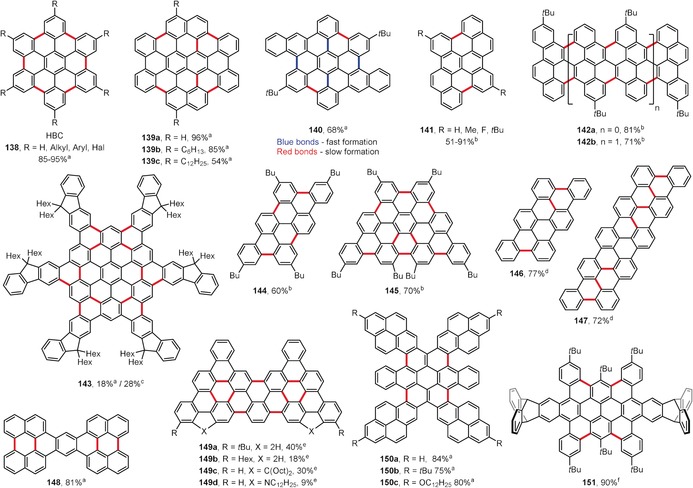
Selected examples of PAHs obtained by intramolecular oxidative coupling. Cyclization conditions: a) FeCl_3_, CH_2_Cl_2_, CH_3_NO_2_, RT; b) DDQ, TfOH, CH_2_Cl_2_, 0 °C; c) DDQ, MsOH, CH_2_Cl_2_, 0 °C; d) FeCl_3_, CH_2_Cl_2_, 0 °C (**146**) or RT (**147**); e) FeCl_3_, CH_2_Cl_2_, 4 Å MS, 0 °C; f) FeCl_3_, DDQ, CH_2_Cl_2_, RT.

One of the most important examples is hexa‐*peri*‐hexabenzocoronene (HBC, **138**, R=H; Figure 10). Although HBC and its derivatives had been obtained previously, the yields were typically very low, and it was not until Müllen and co‐workers developed the Cu^II^/AlCl_3_ or FeCl_3_‐promoted oxidation of hexaphenylbenzenes that HBC derivatives could be obtained in good yields.^36^ The latter conditions in particular, where FeCl_3_ is dissolved in nitromethane prior to its addition to a solution of the precursor in dichloromethane, provide the best yields for the broadest scope of HBCs (**138**; Figure 10),^35^, ^122^, ^123^ which opened the door to the synthesis of large PAHs and nanographenes.

Figure 10 depicts some recently reported large PAHs that were synthesized under intramolecular oxidative coupling conditions. Tetrabenzocircumpyrene derivatives **139 a**–**c** have been synthesized in good yields using FeCl_3_ as an oxidant in a CH_2_Cl_2_/CH_3_NO_2_ mixture.^124^

Compound **140** is an extended hexabenzocoronene that was obtained by Dichtel and co‐workers.^125^ The authors observed that the formation of the first four bonds is very fast (Figure 10, blue bonds), thereby providing an isolable partly fused, twisted intermediate, which after prolonged exposure to the FeCl_3_ oxidant furnishes the final HBC derivative in good yield.

In addition to FeCl_3_, DDQ in the presence of Brønsted acids (Rathore conditions) is also frequently used in the synthesis of PAH molecules by oxidative cyclization. Compounds **141**–**145** are examples of PAHs synthesized using DDQ as an oxidant in the presence of triflic or methanesulfonic acid (Figure 10).^126^, ^127^, ^128^, ^129^ In the case of the hexafluorenyl‐fused HBC **143**, DDQ/MsOH proved to be more efficient than FeCl_3_ (28 % versus 18 % yield).^128^ As a consequence of the presence of twelve hexyl chains pointing out of the PAH plane, the aggregation of **143** in solution is restricted and it shows good solubility in common organic solvents.

Murakami, Itami, and co‐workers have recently developed an interesting method for the palladium‐catalyzed annulative dimerization of monochlorinated oligo‐*para*‐phenylenes to afford substituted triphenylenes.^130^ The products contain (oligo)phenylene residues in a parallel orientation. Two of these triphenylene derivatives have been subjected to oxidative cyclization conditions with FeCl_3_ in dichloromethane to give the corresponding products **146** and **147** in good yields (Figure 10). The analogous oxidative coupling of phenylene residues was not possible when two linear oligophenylene moieties were connected by only one covalent bond, thus indicating that the triphenylene core is necessary to hold the residues in a parallel orientation to enable the reaction to proceed efficiently. However, the analogous reaction was reported to be successful for a singly bonded tri‐*para*‐phenylene dimer when the subunits were end‐capped with octyloxy substituents,^131^ thereby furnishing the tetraoctyloxy derivative of **147** in good yield. A similar approach towards triphenylene derivatives, but employing oligo‐*para*‐phenylene iodides instead of chlorides, was independently developed by Shi and co‐workers; the products also underwent oxidative planarization upon treatment with FeCl_3_.^132^

Tao and co‐workers have utilized the FeCl_3_/CH_3_NO_2_/CH_2_Cl_2_ system for oxidative cyclization reactions in the synthesis of numerous extended planar and curved PAHs.^133^, ^134^, ^135^, ^136^ Tetranaphthopentacene **148** is one of many interesting examples prepared by this research group (Figure 10).^135^

Roberts, Krische, and co‐workers have exploited ruthenium‐catalyzed diol‐diene benzannulation for the construction of various polyphenylene‐type PAH precursors.^137^ Treatment of zigzag‐type oligophenylene precursors with FeCl_3_ in dichloromethane in the presence of 4 Å molecular sieves led to the formation of nine new C_aryl_–C_aryl_ bonds and afforded PAHs **149 a**–**d** in moderate yields (9–40 %; Figure 10). The presence of nitrogen bridges in **149 d** significantly decreased the reaction efficiency.

Müllen, Chen, and co‐workers have reported the synthesis of a series of tetrapyrene‐fused benzocoronenes **150 a**–**c** (Figure 10).^138^ All compounds were obtained in very good yields by the FeCl_3_‐mediated oxidation of the corresponding tetrapyrenylpentacene‐quinodimethanes.

Mastalerz and co‐workers reported the synthesis of triptycene‐flanked tetrabenzoovalene **151**, which was obtained in 90 % yield upon oxidation with a FeCl_3_/DDQ mixture (Figure 10).^139^ As a result of the large steric hindrance, the whole system is contorted and easily loses the two central *tert*‐butyl substituents under acidic conditions.

In addition to the structures shown in Figure 10, many other PAHs have been synthesized using the oxidative coupling method^140^, ^141^, ^142^, ^143^, ^144^, ^145^, ^146^, ^147^ which, due to space limitations, cannot be included in this Review.

**Scheme 21:**
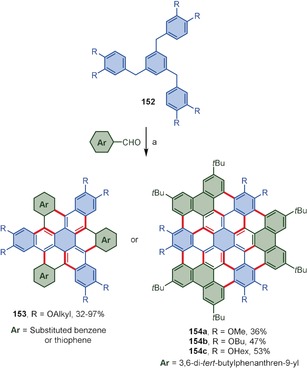
Reaction conditions: a) FeCl_3_ (10 mol %), Ac_2_O, CH_2_Cl_2_, CH_3_NO_2_, RT, then excess FeCl_3_.

Wei and co‐workers developed a convenient method for the synthesis of threefold symmetrical nanographenes **153** and **154** by reacting 1,3,5‐tribenzylbenzene derivatives **152** with aromatic aldehydes in the presence of FeCl_3_ as a catalyst/oxidant and acetic anhydride as a dehydrant in a CH_2_Cl_2_/CH_3_NO_2_ mixture (Scheme 21).^148^ The reaction is a combination of Friedel–Crafts‐type substitution (between formyl groups of aldehydes and aryl rings), dehydrogenative aromatization, and intramolecular oxidative coupling and produces the nanographenes **153** and **154 a**–**c** in good yields.

**Scheme 22:**
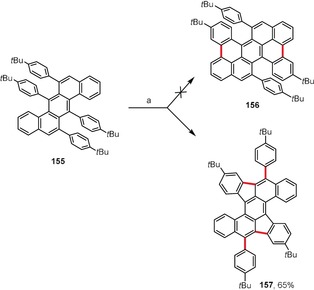
Exemplary dehydrogenative coupling accompanied by a double 1,2‐aryl shift. Reaction conditions: a) DDQ (5 equiv), TfOH, CH_2_Cl_2_, 0 °C.

The in situ 1,2‐aryl shift often changes the course of the oxidative aromatic coupling. Indeed, 1,2‐aryl shifts during the course of oxidative aromatic coupling reactions have been regularly encountered over the last few years.^149^, ^150^ A highly interesting example was published by Müllen and co‐workers during attempts to synthesize tetrabenzo[*a,cd,j,lm*]perylene **156** by the Scholl reaction of 6,7,13,14‐tetraphenylbenzo[*k*]tetraphene **155** (Scheme 22).^151^ Instead of **156**, compound **157** was obtained as a result of two 1,2‐aryl shifts. Müllen and co‐workers showed that a radical cation mechanism is much more likely for the first 1,2‐aryl shift. In the following study, the authors utilized the discovered rearrangement in the synthesis of fused dibenzo[*a*,*m*]rubicene, a bowl‐shaped subunit of C_70_ fullerene.^152^

**Scheme 23:**
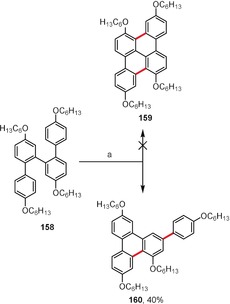
Reagents and conditions: a) FeCl_3_ (7.3 equiv), CH_3_NO_2_, CH_2_Cl_2_, RT.

Another example of this trend has been presented by Hartley and co‐workers, who have shown that terphenyl **158** undergoes single oxidative coupling and a 1,2‐aryl shift to afford **160** and not hexacycle **159** (Scheme 23).^153^

**Scheme 24:**
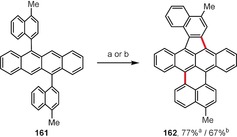
Reagents and conditions: a) FeCl_3_ (16 equiv), CH_2_Cl_2_, CH_3_NO_2_, RT; b) DDQ (2.4 equiv), TfOH, CH_2_Cl_2_, RT.

Under oxidative coupling conditions (FeCl_3_ or DDQ), acenes bearing aryl groups attached to the central benzene rings generally cyclize to form new five‐membered rings.^142^, ^144^ Scheme 24 depicts an especially interesting example, where oxidation of symmetric tetracene **161** led to the unsymmetric product **162** containing two new rings of different sizes: five‐ and six‐membered.^154^

**Figure 11:**
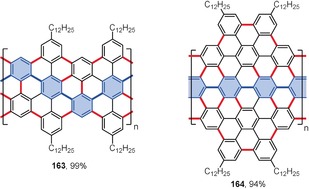
Examples of graphene nanoribbons. Cyclization conditions: FeCl_3_ (excess), CH_2_Cl_2_, CH_3_NO_2_, RT. The benzene rings constituting the main polyphenylene polymer chain are marked in blue.

The bottom‐up synthesis of carbon nanoribbons is yet another impressive demonstration of the prowess of intramolecular oxidative coupling. Belonging to the nanographene class, carbon nanoribbons are ribbon‐ or tape‐like graphene fragments which are highly interesting because of their promising optoelectronic and semiconducting properties. Their syntheses have been extensively reviewed recently.^5^, ^155^, ^156^ Figure 11 presents the structures of the two exemplary carbon nanoribbons **163** 157 and **164** 158 obtained in excellent yields by Feng, Müllen, and co‐workers from the corresponding polymeric precursors using FeCl_3_ as an oxidant in a CH_2_Cl_2_/CH_3_NO_2_ mixture. The precursor for nanoribbon **163** was obtained by Diels–Alder polymerization^159^ of an appropriate acetylene‐functionalized tetraarylcyclopentadienone monomer. The weight‐average molecular weight (*M*_w_) of the resulting polymer reached 640 kg mol^−1^. FeCl_3_‐promoted planarization of the polymer furnished the nanoribbon with a cove‐type edge structure and width of 0.7–1.1 nm.^157^ The poly‐*para*‐phenylene scaffold of the precursor for nanoribbon **164** was constructed by Suzuki polymerization.^158^ After treatment with FeCl_3_, the necklace‐like nanoribbon **164** was obtained in high yield.

Recent reports show that large PAHs, such as HBC, triangular C_60_H_42_, or even the hexagonal graphene plate C_222_H_150_, can be successfully synthesized from the corresponding branched polyphenylene precursors in a mechanochemical fashion by grinding with FeCl_3_ in a planetary ball mill.^160^

### 3.2 Dyes and Heterocyclic Polyarenes

The intramolecular oxidative coupling of arenes typically results in a connection of two aromatic rings in a coplanar orientation, which elongates the conjugated system of π‐electrons and usually strongly affects the photophysical properties of the obtained polycyclic aromatic products. Hence, it is not surprising that this method has been widely used for π‐expansion in various organic chromophores and fluorophores.

**Figure 12:**
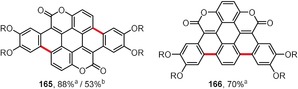
Conditions: a) FeCl_3_ (10 equiv), cat. BF_3_⋅Et_2_O, CH_2_Cl_2_, RT; b) *hν* (365 nm), air, THF, RT. R=Hexyl.

Coumarins are bright organic fluorophores with multiple applications in fluorescence imaging.^161^ Figure 12 presents the structures of π‐expanded coumarins obtained by the Scholl reaction or by intramolecular oxidative coupling. The pentacene‐based coumarin dimer **165** 162 and its regioisomer **166** 163 can both be efficiently synthesized from the corresponding precursors through an FeCl_3_‐promoted intramolecular oxidative coupling in the presence of a catalytic amount of BF_3_⋅Et_2_O. Compound **165** can also be obtained by a photochemical cyclization; however, the yield of the transformation was considerably lower than the oxidation with FeCl_3_.^162^

**Scheme 25:**
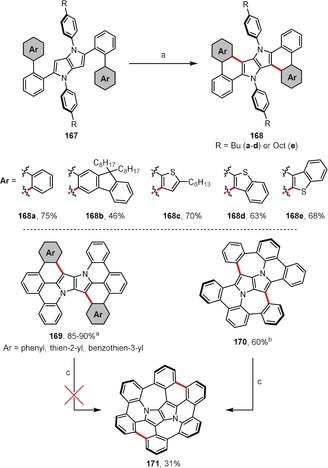
FeCl_3_‐mediated π‐expansions of pyrrolo[3,2‐*b*]pyrroles. Cyclization conditions: a) FeCl_3_ (20 equiv), CH_2_Cl_2_, CH_3_NO_2_, RT; b) FeCl_3_ (20 equiv), DCE, CH_3_NO_2_, 80 °C; c) Au(111), 320 °C.

Pyrrolo[3,2‐*b*]pyrrole is the most electron‐rich among the known neutral 10π‐aromatic systems.^164^ This heterocycle recently gained more attention because of the discovery of an efficient one‐step, multicomponent synthesis of 1,2,4,5‐tetraarylpyrro[3,2‐*b*]pyrrole dyes with strong fluorescence. By using this method, 2,5‐di(biaryl)‐substituted derivatives **167** have been obtained from the reaction of *ortho*‐arylbenzaldehyes with 4‐alkylanilines and diacetyl (Scheme 25).^165^ These products readily react with FeCl_3_ to give the series of π‐expanded pyrrolopyrroles **168 a**–**e**. Gryko and co‐workers devoted much effort to the synthesis of the bowl‐shaped pyrrolopyrrole **171**, which would have an inverse Stone‐Thrower‐Wales^166^, ^167^ topology (Scheme 25). To meet this goal, compounds **169** and **170** were prepared as precursors for **171**, with FeCl_3_ used to efficiently close the six‐membered and seven‐membered rings, respectively.^168^, ^169^ Unfortunately, all attempts to convert these precursors into **171** by means of classic organic synthesis failed. Finally, however, compound **171** was generated on an Au(111) surface by thermal annealing of the precursor **170** under ultrahigh vacuum (see Section 4).^169^

**Scheme 26:**
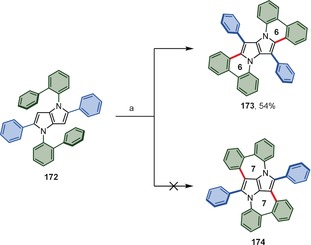
Reagents and conditions: a) FeCl_3_ (20 equiv), DCE, CH_3_NO_2_, 80 °C.

Gryko and co‐workers revealed the first case of a 1,2‐aryl shift for aromatic heterocycles (Scheme 26).^170^ Tetraarylpyrrolo[3,2‐*b*]pyrrole (TAPP) **172** possessing [1,1′‐biphenyl]‐2‐yl substituents attached to the pyrrolic nitrogen atoms undergoes selective double dehydrogenative cyclization accompanied by a twofold 1,2‐aryl shift under oxidative aromatic coupling conditions. As a result, instead of the expected product **174** possessing two seven‐membered rings, dye **173** is formed.

**Scheme 27:**
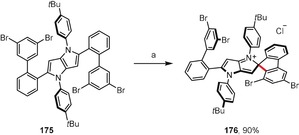
Intramolecular oxidative coupling of pyrrolo[3,2‐*b*]pyrroles. Reagents and conditions: a) FeCl_3_ (20 equiv), CH_2_Cl_2_, CH_3_NO_2_, RT.

Treatment of bulky tetrabromo‐substituted tetraarylpyrrolo[3,2‐*b*]pyrrole **175** with FeCl_3_ gave the spirocyclic cationic fluorene derivative **176** instead of the expected product with new six‐membered rings (Scheme 27).^171^ Computational studies revealed that, in the case of **175**, the formation of the spiro‐five‐membered ring is preferred in terms of both the transition‐state energy and the relative energy of the product. Steric hindrance is undoubtedly the main reason for the different reactivity of compound **175** compared to its analogues that lack additional substituents on the peripheral benzene rings (e.g. **167**; see Scheme 25). In a broader context, these two studies have shown that a C−C bond can form at an already occupied carbon atom if the highest electron density is located there.

Intramolecular oxidative coupling has also been used as a convenient procedure for the ring fusion of perylene diimides (PDI),^172^, ^173^, ^174^ as well as for π‐expansions of BODIPY and aza‐BODIPY‐based dyes,^175^, ^176^, ^177^, ^178^, ^179^ which has led to significant bathochromic shifts of the absorption and emission maxima. A library of π‐expanded imidazoles has been prepared by employing a PIFA/BF_3_⋅Et_2_O system for the oxidative cyclization of variously substituted 1,2,4,5‐tetraarylimidazole derivatives.^180^

**Figure 13:**
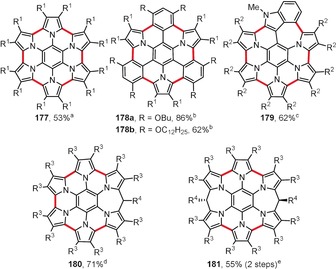
Hexapyrrolocoronene analogues and related compounds. Cyclization conditions: a) FeCl_3_ (36 equiv), CH_2_Cl_2_, CH_3_NO_2_, RT, then N_2_H_4_; b) FeCl_3_ (38 equiv), CH_2_Cl_2_, CH_3_NO_2_, RT; c) BAHA (12 equiv), Et_2_O/THF, RT, then Zn amalgam; d) DDQ (6 equiv), CH_2_Cl_2_, RT, then N_2_H_4_; e) i. DDQ (5 equiv), CH_2_Cl_2_, RT, then HBF_4_/H_2_O, ii. NaBH_4_, THF. R^1^=4‐(trifluoromethyl)phenyl, R^2^=4‐chlorophenyl, R^3^=4‐butoxyphenyl, R^4^=4‐nitrophenyl.

As a consequence of their high electron density, pyrrole‐based compounds are often cumbersome substrates for reactions involving strong oxidants and acids.^181^ However, the efficient intramolecular coupling of pyrrole rings can be achieved under carefully optimized conditions.^182^ A prominent example was provided in 2007 by Müllen and co‐workers, who synthesized the rim‐fused hexapyrrolylbenzene **177** (Figure 13).^183^ The electron‐rich nature of pyrrole was evident by the fact that the FeCl_3_‐mediated cyclization initially led to an overoxidized dicationic form of **177**, which could be reduced to the neutral **177** with hydrazine. More recently, the authors have extended this synthetic method to include analogues of **177** with one, two, or three pyrrole moieties replaced by benzene units (e.g. **178**).^184^

Stępień and co‐workers have synthesized an analogue of **177** with one pyrrole ring replaced by an indole unit (**179**; Figure 13).^185^ The cyclization was accomplished using tris(4‐bromophenyl)ammoniumyl hexachloroantimonate (BAHA, also known as “Magic Blue”), a radical cation oxidant. Similar to **177**, the oxidation leads to an overoxidized aromatic dication of **179** (62 % yield), which after reduction with zinc amalgam affords the neutral **179** quantitatively. The same research group also reported the DDQ‐mediated synthesis of compounds **180** and **181**, expanded derivatives of **177** containing one or two methylene bridges in the outer hexapyrrole rim.^186^ Another spectacular case of multiple intramolecular oxidative aromatic coupling involving eight pyrrole units to form a twisted aza‐PAH containing two unusual N‐doped heptagons was revealed by Uno and co‐workers.^187^

**Figure 14:**
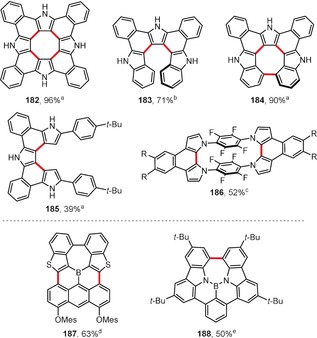
A selection of pyrrole‐based and boron‐doped heterocyclic polycyclic arenes obtained by oxidative cyclization. Reaction conditions: a) DDQ (5 equiv), Sc(OTf)_3_ (5.6 equiv), toluene, reflux; b) PIFA (2.5 equiv), CH_2_Cl_2_, −78 °C; c) DDQ (2 equiv), Sc(OTf)_3_ (7 equiv), CH_2_Cl_2_, RT; d) FeCl_3_ (8 equiv), CH_2_Cl_2_, CH_3_NO_2_, RT; e) DDQ (2.5 equiv), TfOH, CH_2_Cl_2_, 0 °C.

Osuka and co‐workers reported yet another example of an efficient intramolecular oxidative coupling of pyrrole rings (Figure 14). Tetrapyrrole[8]circulene analogue **182** was prepared by employing a so‐called “fold‐in” strategy,^188^ which is a method developed earlier by Stępień and co‐workers to construct strained macrocycles by contraction of the larger, less‐strained precursors.^189^, ^190^ Here, a macrocycle consisting of four pyrrole rings connected by *ortho*‐phenylene moieties was oxidatively contracted to **182** in excellent yield by the action of DDQ and scandium(III) triflate in boiling toluene. Products **183**–**185** were prepared in a similar way.^191^ Depending on the conditions and oxidant used, the bis(indolyl)helicene **183** or the closed, nonplanar circulene analogue **184** can be obtained in good yields from the same precursor. The eight‐membered ring in the latter case readily forms under the same conditions as those used for the synthesis of circulene **182**, whereas the preparation of its open form **183** requires the use of much milder conditions (PIFA in CH_2_Cl_2_ at −78 °C). The efficiency of the DDQ/Sc(OTf)_3_ system for pyrrole–pyrrole intramolecular oxidative coupling has also been demonstrated by the synthesis of paracyclophane **186**, which contains a segregated donor‐acceptor‐donor system with stacked tetrafluorobenzene rings as acceptors.^192^

Boron‐doped polycyclic arenes are gaining more and more attention because of their promising properties and use in organic electronics, light‐emissive materials, and fluorescent sensors.^193^, ^194^ Yamaguchi and co‐workers have synthesized the first planarized, fully conjugated triarylborane **187** using FeCl_3_‐mediated cyclization, which led to the formation of two new bonds between the benzothienyl substituents and an anthracene moiety (Figure 14).^195^ The same group has also used FeCl_3_ as an oxidant in the synthesis of a larger PAH containing two boron atoms.^196^

The polycyclic arene **188** doped with a nitrogen‐boron‐nitrogen unit has been synthesized by using DDQ in the presence of triflic acid to efficiently close the seven‐membered ring (Figure 14).^197^

Many π‐extended porphyrins have been synthesized by inter‐ or intramolecular oxidative coupling; however, they will not be described here due to space restrictions and the fact that this subject has been reviewed recently.^198^, ^199^

**Scheme 28:**
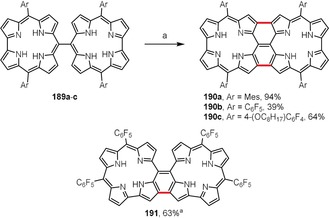
Synthesis of fused corrole dimers. Reaction conditions: a) DDQ (3–3.5 equiv), CHCl_3_, reflux.

As reported by Tanaka, Kim, Osuka, and co‐workers, similar to porphyrin analogues, *meso*‐*meso*‐linked corrole dimers **189 a**–**c** bearing various aryl substituents can also be oxidatively planarized to the corresponding triply linked products **190 a**–**c** (Scheme 28).^200^ The reaction was accomplished using DDQ in boiling chloroform and required high dilution of the starting materials to minimize unwanted intermolecular coupling reactions. The cyclization was accompanied by overoxidation, thereby leading to the loss of one NH hydrogen atom in each corrole macrocycle, which as a consequence became non‐aromatic. Similar overoxidation also occurred in the case of the doubly linked corrole dimer **191**, which was synthesized under similar conditions.^201^ The aromaticity of the corrole ring in both types of dimers, **190** and **191**, can be restored by reduction with NaBH_4_; however, they slowly reconvert back to the stable non‐aromatic forms in air.

### 3.3 Curved, Twisted, and Strained Structures

Nonplanar, curved, and twisted polycyclic aromatic architectures have attracted considerable attention because of their unusual geometries, strained structures, and interesting photophysical and electrochemical properties, which are significantly different from those of their planar analogues.^202^ The synthesis of curved PAHs remains challenging because of the large strain. Nevertheless, recent years have seen a proliferation of curved or twisted PAHs synthesized by the intramolecular oxidative coupling method.

**Figure 15:**
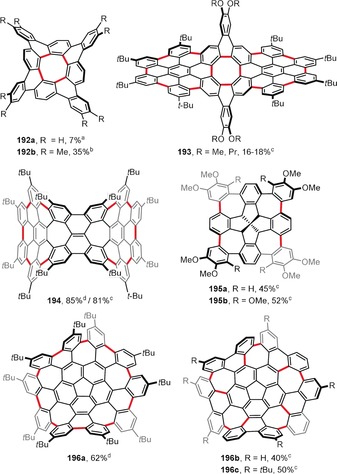
Saddle‐shaped PAHs. Cyclization conditions: a) Cu(OTf)_2_ (24 equiv), AlCl_3_ (24 equiv), CS_2_, 30 °C; b) FeCl_3_ (16 equiv), CH_2_Cl_2_, −20 °C; c) DDQ (1–4 equiv/bond), TfOH, CH_2_Cl_2_, 0 °C (**193**, **195 a**,**b**, **196 b**,**c**) or RT (**194**); d) FeCl_3_ (excess), CH_3_NO_2_, CH_2_Cl_2_, RT.

Planar PAHs naturally consist mainly of fused six‐membered benzene rings. The introduction of rings of different sizes usually induces curvature to the system. Hence, the presence of five‐membered rings usually leads to bowl‐shaped compounds with positive curvature (e.g. corannulene and C_60_). In contrast, larger seven‐ and eight‐membered rings lead to saddle‐shaped systems with negative curvatures (e.g. [7]circulene). Figure 15 presents the structures of saddle‐shaped PAHs synthesized under intramolecular oxidative coupling conditions.

In 2013, Sakamoto and Suzuki reported an elegant synthesis of tetrabenzo[8]circulenes **192 a** and **192 b** by the oxidation of the corresponding macrocyclic octaphenylene precursors with Cu(OTf)_2_/AlCl_3_ and FeCl_3_, respectively (Figure 15).^203^ This was the second successful reported synthesis of an [8]circulene derivative; the first report preceded it only by a few weeks.^204^ In Section 3.2 we described the synthesis of a tetrapyrrole analogue of **192**, compound **182**, which was obtained by a similar “fold‐in” strategy, but using DDQ/Sc(OTf)_3_ as an oxidant.^188^ In contrast to **182**, the products **192 a**,**b** are saddle‐shaped, as evidenced by X‐ray crystallography. By using the same approach, Miao and co‐workers recently synthesized a much larger derivative of [8]circulene, a saddle‐shaped PAH **193** flanked by two fused HBC moieties (Figure 15).^205^ DDQ in TfOH/CH_2_Cl_2_ enabled the formation of all required 14 C_aryl_–C_aryl_ bonds in one step, thereby providing **193** in 16–18 % yield. An alternative method to construct the tetrabenzo[8]circulene scaffold starts with tetraphenyl‐substituted tetra‐*ortho*‐phenylenes which already contain a central eight‐membered ring. These phenylenes are treated with DDQ/TfOH to effect the oxidative closure of four external benzene rings.^206^ This approach is efficient (47–72 % yield) for the synthesis of variously substituted derivatives of tetrabenzo[8]circulene that can undergo further functionalization.

The dipleiadiene‐embedded PAH‐saddle **194** contains two seven‐membered rings embedded within two fused [7]circulene moieties (Figure 15). Its synthesis has been realized by Miao and co‐workers in high yields using FeCl_3_ and DDQ as oxidants.^207^ The same group also synthesized other heptagon‐containing saddle‐shaped PAHs using intramolecular oxidative coupling in key steps.^208^, ^209^

Kuck, Chow, and co‐workers have utilized the DDQ/TfOH oxidative system for the synthesis of molecules **195 a** and **195 b** bearing tetracoordinated carbon atom cores within saddle‐shaped *o*,*p*,*o*,*p*,*o*,*p*,*o*,*p*‐cyclooctaphenylene belts (Figure 15).^210^ Despite large strain, both products were obtained in relatively good yields of about 50 %. The authors have also recently reported the synthesis of enantiopure isomers of **195 b**.^211^

An oxidative closure of five seven‐membered rings around a corannulene core yields the large asymmetric saddle‐shaped PAHs **196 a**–**c**, as reported by Itami, Scott, and co‐workers (Figure 15).^212^, ^213^, ^214^ The authors demonstrated two different oxidative coupling strategies to construct the curved skeleton of these compounds. Oxidation of deca(4‐*tert*‐butylphenyl)corannulene with FeCl_3_ afforded the product **196 a** in 62 % yield,^212^ whereas penta(biphenyl)‐substituted corannulenes yielded the corresponding curved PAHs **196 b** and **196 c** upon treatment with DDQ/TfOH (40 and 50 % yield, respectively).^212^, ^213^, ^214^ The ease of closure of the seven‐membered rings is noteworthy, since an analogous reaction failed for the model compound [6]helicene.^212^ Further functionalization of **196 c** by C−H borylation and introduction of polar groups by Suzuki coupling provided a water‐soluble warped nanographene which could be used to promote photoinduced cell death in living HeLa cells.^214^ The authors have also reported an analogue of **196** with five benzene rings replaced by thiophene rings.^215^

**Figure 16:**
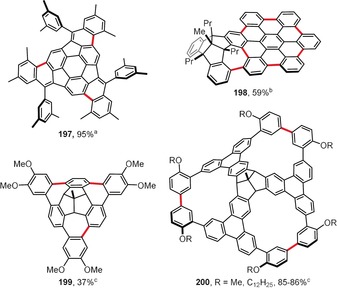
Bowl‐shaped compounds. Cyclization conditions: a) DDQ (6 equiv), Sc(OTf)_3_, CH_2_Cl_2_, RT; b) Cu(OTf)_2_ (42 equiv), AlCl_3_ (43 equiv), CS_2_, 45 °C; c) DDQ (4.5–5 equiv), TfOH, CH_2_Cl_2_, 0 °C.

Figure 16 presents four examples of bowl‐shaped PAHs synthesized using intramolecular oxidative coupling in the final step. Compound **197** is a derivative of hemifullerene (C_60_ half), which was obtained in excellent yield and regioselectivity by Amaya, Ito, and Hirao through DDQ/Sc(OTf)_3_‐promoted oxidative cyclization of the precursor derived from the condensation of sumanene with three molecules of benzophenone.^216^ Mughal and Kuck synthesized the tribenzotriquinacene‐HBC hybrid **198** using the classical Müllen oxidative coupling conditions by employing copper(II) triflate/aluminum chloride in CS_2_ (Figure 16).^217^ The product contains one seven‐membered ring formed between the HBC and one of the benzene rings in the triquinacene core. Attempts to synthesize a *C*_3_‐symmetric analogue of **198** containing three HBC moieties failed.^218^

Kuck, Chow, and co‐workers have, however, reported the syntheses of other bowl‐shaped derivatives of tribenzotriquinacene bearing threefold symmetry: compounds **199** 219, 220 and **200** 221 (Figure 16). Triquinacene **199** was obtained in 37 % yield from an unsymmetric triaryltribenzotriquinacene precursor by oxidative closure of three seven‐membered rings upon treatment with DDQ/TfOH. The product **199** contains an *o*,*p*,*o*,*p*,*o*,*p*‐cyclohexaphenylene belt at the rim of the bowl. Even more interesting is the synthesis of derivative **200**, a rare example of oxidative macrocyclization, which was achieved using similar conditions as for **199**, however, in a much higher yield (ca. 85 %; Figure 16). The high efficiency of the threefold macrocyclization can be attributed to the rigid framework of the tris(triphenyleno)triquinacene system, which spatially prearranges the alkoxyphenyl groups for intramolecular oxidative coupling.

Smith and Lucas have reported the synthesis of interesting tetraarylene‐bridged cavitands based on resorcin[4]arene using FeCl_3_ or DDQ as the oxidants.^222^

Helicenes have fired researchers’ imagination ever since the first synthesis and resolution of [6]helicene in 1956.^223^ The synthesis of helicenes has long been dominated by iodine‐catalyzed photocyclizations of stilbene derivatives.^224^, ^225^ Numerous examples published in recent years clearly show that the intramolecular oxidative coupling can be no worse, or even superior, for the construction of strained helicene scaffolds, especially for products containing multiple helicene fragments.

**Figure 17:**
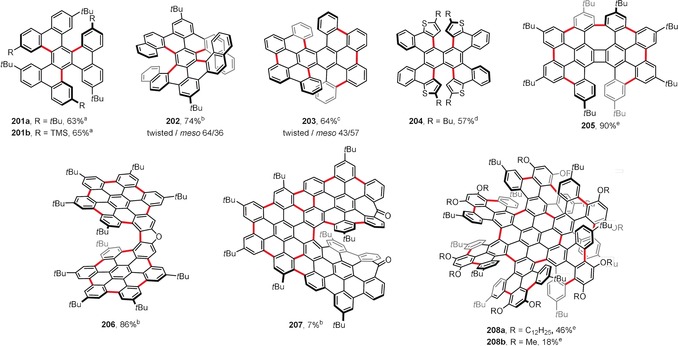
Helicenes. Cyclization conditions: a) FeCl_3_ (20 equiv), CH_2_Cl_2_, CH_3_NO_2_, RT; b) DDQ (1.0–1.2 equiv/bond), TfOH, CH_2_Cl_2_, 0 °C; c) DDQ (7 equiv), DCE, TfOH, 0 °C; d) MoCl_5_ (10 equiv), CH_2_Cl_2_, 4 Å MS, 0 °C; e) DDQ (1.1–2.0 equiv/bond), MsOH, CH_2_Cl_2_, 0 °C. For all products obtained as mixtures of enantiomers/diastereoisomers, the structure of one selected isomer is shown.

The pioneering synthesis of dibenzo[5]helicene by Durola and co‐workers using FeCl_3_ as the oxidant indicated that intramolecular oxidative coupling can be a very efficient method for the preparation of helicenes.^226^ By using this strategy the authors have synthesized in high yields the tris[5]helicenes **201 a** 226 and **201 b** 227 (Figure 17), as well as many other distorted PAHs with embedded helicene moieties.^228^ Many reports of oxidatively synthesized helicenes soon followed—selected examples are shown in Figure 17.

Müllen, Narita, and co‐workers reported that, instead of the expected perihexacene derivative, the reaction of the tetranaphthyl‐substituted precursor with DDQ/TfOH produced the double [7]helicene **202** in 74 % yield as a mixture of diastereoisomers (a pair of twisted enantiomers and a *meso* form, Figure 17).^229^ Such a regioselectivity of the cyclization is impressive, since [7]helicene moieties are even more strained than [5]helicenes. Recently, an analogue of **202** with two [6]helicene parts has also been described by using oxidative coupling in the final step.^230^ Moreover, Miao and co‐workers described a similar double helicene with a PAH skeleton regioisomeric to **202**.^231^

Itami and co‐workers synthesized a double [6]helicene **203** by the reaction of tetra(biphenyl)‐substituted naphthalene with DDQ/TfOH in dichloroethane (Figure 17).^232^ Similar to **202**, the product was also obtained as a mixture of three diastereoisomers. An analogous reaction of a thiophene‐based precursor with MoCl_5_ in dichloromethane over 4 Å molecular sieves afforded the not fully cyclized tetrahelicene **204** instead of the expected double helicene.^233^ The formation of **204** was kinetically controlled; upon heating, the propeller‐like arrangement of its thiophene‐containing wings underwent quantitative isomerization to the less‐strained diastereoisomer with the wings alternately tilted “up” and “down” relative to the central naphthalene plane. Repeating the reaction with MoCl_5_ at room temperature without the addition of molecular sieves led to the formation of the two remaining C_aryl_−C_aryl_ bonds, thereby providing an analogue of **203** (double helicene) containing four chlorine atoms as a consequence of the MoCl_5_‐mediated chlorination.^234^

Octa(4‐*tert*‐butylphenyl)biphenylene undergoes efficient oxidation with DDQ/MsOH to form the distorted PAH **205**, which is based on a [7]helicene moiety with one four‐membered ring (Figure 17).^235^ Interestingly, one twisted eight‐membered ring was also formed in this reaction.

The remaining three compounds presented in Figure 17 (**206**–**208**) were all reported in 2018 and belong to the class known as “superhelicenes”, a term coined for unusually large helical PAHs. Here the synthetic power of intramolecular oxidative coupling is fully unleashed, as almost all members of this class are prepared using the oxidative coupling method.

Compound **206** was synthesized by Jux and co‐workers using DDQ/TfOH to oxidatively close 13 new C_aryl_–C_aryl_ bonds in one operation (Figure 17).^236^ **206** consists of two penta(*tert*‐butyl‐HBC) units fused to a central furan core, hence, forming an oxa[7]helicene derivative. Replacing DDQ by FeCl_3_ as an oxidant leads to the non‐fully cyclized bis‐HBC ether (lacking the furan ring) which, however, readily cyclizes to **206** upon exposure to light.

Campaña and co‐workers synthesized the superhelicene **207** in 7 % yield by employing DDQ/TfOH as an oxidant (Figure 17).^237^ The authors have also reported the synthesis of other large PAH‐helicenes and superhelicenes by oxidative coupling.^238^, ^239^

Compounds **208 a**, **208 b**,^240^ and larger analogues^241^ reported by Wang and co‐workers are amongst the most impressive examples of curved PAH derivatives prepared by oxidative coupling (Figure 17). These propeller‐shaped hexapole [7]helicenes based on a HBC core were obtained by oxidation of the corresponding dendrimer‐like oligophenylene precursor with DDQ/MsOH, which resulted in the formation of 18 new C_aryl_–C_aryl_ bonds in one step. The enantiomers of **208 a** were separated by HPLC on a chiral phase and the helicity of the fractions was elucidated from direct observation with a STM microscope.

**Figure 18:**
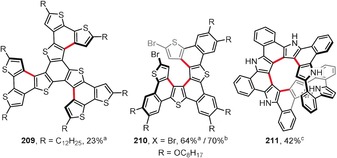
Heterocyclic helicene‐like compounds. Cyclization conditions: a) FeCl_3_ (6.6–12 equiv), CH_2_Cl_2_, CH_3_NO_2_, 0 °C; b) DDQ (4.5 equiv), BF_3_⋅Et_2_O, CH_2_Cl_2_, 0 °C; c) DDQ (5 equiv), Sc(OTf)_3_, toluene, reflux.

Figure 18 presents helicene‐like compounds based on thiophene and pyrrole. Bu using FeCl_3_ as an oxidant, Aida and co‐workers synthesized propeller‐shaped molecules containing multiple thiophene rings, that is, **209** and its two *C*_3_‐symmetric regioisomers.^242^

Heterohelicene **210** was synthesized by Dehaen and co‐workers as a demonstrative application of the oxidative coupling conditions they had optimized for the synthesis of dithienyltriphenylene analogues.^243^ Cyclization using DDQ or FeCl_3_ was found to be much faster than photocyclization (1.5–2 h versus 72 h for completion).

As an extension of the synthesis of the [8]circulene analogue **182**,^188^ Osuka and co‐workers performed a similar reaction for a hexameric analogue, which afforded the non‐fully cyclized product **211** (Figure 18).^244^ Its structure contains a pentaaza[9]helicene moiety with its ends connected through a 2,5‐diphenylpyrrole bridge. An analogous reaction for a macrocyclic hexathiophene counterpart led to a product composed of [9]‐ and [5]helicene segments.

**Figure 19:**
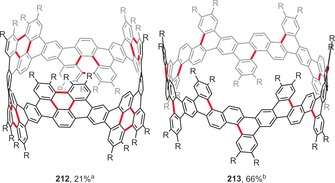
Cyclization conditions: a) FeCl_3_ (100 equiv), CH_2_Cl_2_, −20 °C; b) DDQ (22 equiv), Sc(OTf)_3_, toluene, reflux. R=OPr.

The Müllen^245^, ^246^ and Jasti^247^ research groups tried to apply intramolecular oxidative coupling in the synthesis of segments of carbon nanotubes (so‐called nanobelts); however, their attempts failed because of strain‐relieving rearrangements. Only very recently, Chi, Miao, and co‐workers succeeded in the synthesis of the armchair and chiral carbon nanobelts **212** and **213**, respectively, using FeCl_3_ or DDQ/Sc(OTf)_3_ as the oxidants (Figure 19).^248^ This impressive achievement is a crowning demonstration of the capabilities of the oxidative coupling method. The same group has recently reported the preparation of a thiophene‐based nanoring using FeCl_3_ as the oxidant.^249^

## 4 Surface‐Assisted (Cyclo)Dehydrogenation (CDH)

**Scheme 29:**
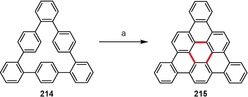
The concept of a surface‐assisted CDH process toward carbon‐rich materials.^262^ Reagents and conditions: a) Cu(111), ca. 230 °C.

In contrast to a typical oxidative aromatic coupling reaction, a surface‐assisted CDH process can be considered a greener alternative because this method generally produces a minimal amount of by‐products and it does not require any solvent. An exemplary process of this type is the formation of tribenzo[*a*,*g*,*m*]coronene (**215**) from its molecular precursor **214** (Scheme 29). Research into the assembly of carbon‐rich materials by an on‐surface method was initiated by the synthesis of fullerene C_60_ and its triaza‐analogue,^250^ hexabenzocoronene (**138**, R=H),^251^ and other aromatic hemispheres^252^ from compounds **138**. Since then, many challenging syntheses of carbon‐based nanostructures have been realized by employing this approach, including single‐chirality (that is, having one single chirality index (*n,m*) indicating the length and the direction of the circumventing vector) single‐walled carbon nanotubes,^253^ the longest to‐date acenes^254^ and periacenes,^255^ PAHs with embedded nonhexagonal rings,^256^ and carbon‐rich molecules with embedded nitrogen atoms.^169^, ^250^, ^257^ An indisputable advantage of this method is the clear preference of the arene rings to undergo exclusively the desired cyclization that leads to the extended nanographenes and results in low‐defect assemblies. Surface‐assisted synthesis has been elegantly summarized in recent literature,^258^, ^259^, ^260^, ^261^ which is why we will only briefly describe the most important contributions to this field in the following section.

**Scheme 30:**
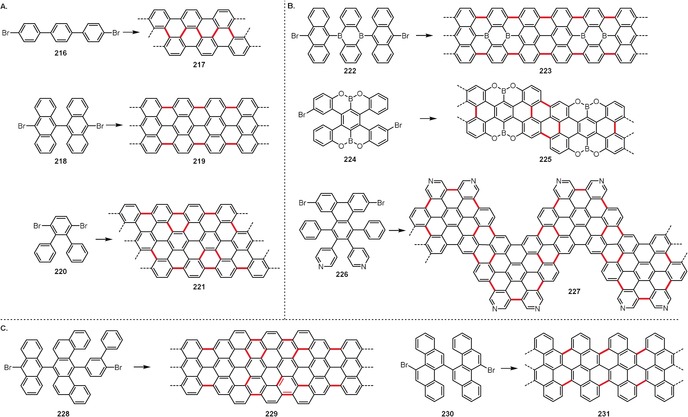
Selected examples for the on‐surface synthesis of atomically precise graphene nanoribbons (GNRs) from small‐molecule precursors.

As a consequence of the excellent physicochemical properties of graphene, a great amount of effort has been made by researchers to prepare atomically precise graphene nanoribbons (GNRs). The general concept for the rational synthesis of such assemblies employing a CDH reaction usually includes two steps: 1) an on‐surface polymerization of a halogenated small‐molecule precursor by a homolytic cleavage of the carbon–halogen bond after evaporation onto a clean noble metal surface under an ultrahigh vacuum and 2) a CDH sequence at elevated temperature, which yields the fully conjugated nanoribbon structure. Following this concept, many atomically precise GNRs have been prepared from the corresponding molecular precursors (Scheme 30; GNR **217**,^263^ **219**,^264^ **221**,^265^ **223**,^266^ **225**,^267^ **227**,^268^ **229**,^269^ and **231** 127). The physicochemical properties of such GNRs may by altered not only by changing the size of the assemblies (Scheme 30 A), but also by doping with heteroatoms (Scheme 30 B) or changing the edge structure of the GNRs (Scheme 30 C).

**Scheme 31:**
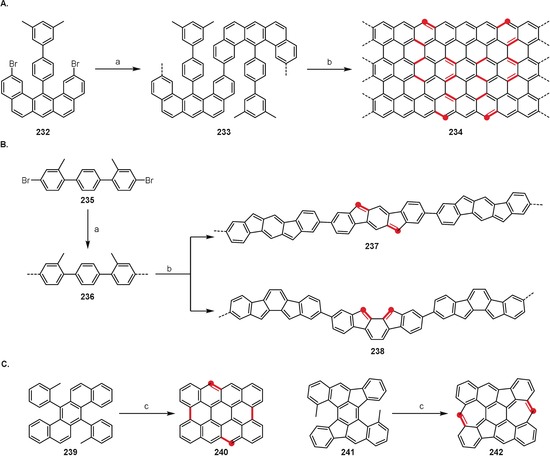
Reagents and conditions: a) Au(111), 200 °C; b) Au(111), 350 °C; c) Au(111), 300 °C. Red dots denote carbon atoms originating from methyl groups.

As has been proved many times, the CDH process occurs typically between two arene rings in proximity. Fasel, Müllen, and co‐workers have found that, during the CDH process, methyl groups that are placed in proximity to aryl rings undergo oxidative cyclization with neighboring aromatic units.^270^ Specifically, the molecular precursor **232** undergoes polymerization followed by a series of CDH processes to afford graphene nanoribbon **234** with a zigzag edge topology (Scheme 31 A). This concept was further extended to dibromodimethylterphenyl **235** (Scheme 31 B),^271^ where the polymerization/surface‐assisted oxidative ring‐closure sequence between a methyl group and the neighboring aryl moiety gives rise to indenofluorene‐based polymers **237** and **238**. This strategy could eventually be employed in the preparation of graphene nanoribbons containing five‐membered rings, because such a modification at specific positions can allow for the fine‐tuning of electronic properties. According to the same strategy, ultra‐low‐gap open‐shell molecules, such as *peri*‐tetracene (**240** from **239**), a benzenoid graphene fragment with a zigzag edge topology, and dibenzo[*a*,*m*]dicyclohepta[*bcde*,*nopq*]rubicene (**242** from **241**), a nonbenzenoid nonalternant structural isomer of *peri*‐tetracene with two embedded azulene units, were synthesized^272^ by surface‐assisted CDH (Scheme 31 C). Spin‐polarized DFT calculations revealed that both compounds should exhibit an open‐shell singlet ground state, thus making them promising candidates for spintronic applications.

**Scheme 32:**
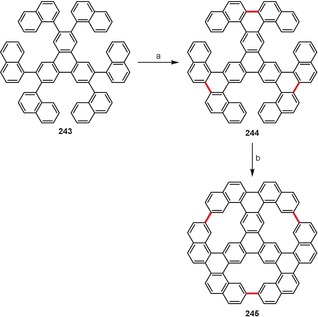
Reagents and conditions: a) DDQ (3.6 equiv), CH_2_Cl_2_/CF_3_SO_3_H (9:1), 0→25 °C, 75 %; b) Au(111), 350 °C.

The power of the CDH reaction has been recently highlighted by Fasel, Müllen, and co‐workers (Scheme 32).^273^ A typical DDQ‐mediated oxidative aromatic coupling performed on **243** leads to the propeller‐shaped molecule **244**. Although a classical oxidative aromatic coupling method was unsuccessful, the CDH process was accomplished on a Au(111) surface at elevated temperature, thus giving the nonplanar porous nanographene analogue **245**.

Despite all the advantages described above, the CDH method still has some limitations. Many functional groups are not stable to the elevated temperatures required and, thus, the possibility of derivatizing the structures prepared by CDH is rather limited. Moreover, the synthesized nanographenic structures may also be unstable under CDH conditions. For practical applications, large amounts of a given material should ideally be obtainable in a relatively short amount of time; however, the CDH method does not yet fulfill this criterion.

## 5 Summary and Outlook

From small‐molecule natural products and drugs, through fluorescent dyes and polycyclic aromatic hydrocarbons to nanographenes, curved arenes, and nanobelts—the wealth of structures that can be synthesized by the oxidative coupling of arenes is tremendous. Enormous efforts by many researchers, from both theoretical and experimental standpoints, has led to an improved understanding of oxidative coupling and provided definitive evidence for the two main mechanisms: via arenium and radical cation intermediates. Slowly, predominantly thanks to the studies by the groups of Waldvogel, Müllen, and Pappo, we are approaching the point where it is possible to predict the reaction outcome before the reactants are even put into the flask; however, a lot of research is still required to reach this goal.

Simultaneously to the mechanistic studies, significant progress has been made in the development of new reagents and conditions, in particular those enabling the more efficient and more selective intermolecular oxidative homo‐ and cross‐coupling of arenes. The most interesting of these include MoCl_3_[OCH(CF_3_)_2_]_2_, (*t*BuO)_2_, Rh/C, and AgSO_4_. However, in many cases of cross‐coupling a large excess of one arene is still necessary to achieve good yields of heterobiaryls. The synthetic possibilities have also been vastly expanded by the broader application of electrochemical methods.

In the meantime, the intramolecular oxidative coupling of arenes has revealed its full synthetic potential, which can be concluded from the vast number of different structures that have been synthesized in recent years. Compared to the intermolecular version, the regioselectivity of intramolecular oxidative coupling is much easier to predict and plan. Moreover, many of the structures that are accessible by intramolecular oxidative coupling, in particular large planar and curved PAHs, would be nearly impossible to synthesize using other methods for forming biaryl bonds because of the required prefunctionalization. Although a variety of valuable reactants are being developed for oxidative coupling, intramolecular oxidative coupling is dominated by two oxidants: FeCl_3_ and DDQ/acid (Rathore conditions). These two reagents have been used by the Müllen, Itami, Miao, and Wu groups for the preparation of the most spectacular PAH molecules to date, including nanographenes, superhelicenes, and nanobelts. It is also worth emphasizing that some very strained products, such as helicenes, bowls and saddles, can be synthesized using this method. Indeed, in contrast to earlier assumptions, the Scholl reaction is capable of proceeding even if ring closure is accompanied by the introduction of significant steric strain that leads to nonplanar molecules.^274^, ^275^ Progress has been particularly impressive in the case of surface‐assisted dehydrogenation, which has enabled the synthesis of nanographenes of unprecedented size by scanning tunneling microscopy.^169^, ^276^

Despite all the impressive progress summarized herein, methods for the oxidative coupling of arenes still have some limitations, which will no doubt be the focus of future studies.

## Conflict of interest

The authors declare no conflict of interest.

## Biographical Information

*Marek Grzybowski obtained his PhD from the Institute of Organic Chemistry of the Polish Academy of Sciences in 2014 under the supervision of Prof. D. T. Gryko. He then carried out postdoctoral research under a JSPS fellowship in the group of Prof. S. Yamaguchi at Nagoya University. Currently he is an assistant professor at the Institute of Organic Chemistry of the Polish Academy of Sciences. His research focuses on the development of photostable fluorophores for bioimaging and the synthesis of extended π‐systems and strained polycyclic arenes*.

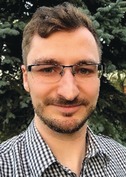

## Biographical Information

*Bartłomiej Sadowski was born in 1990 in Nowy Dwór Mazowiecki*, *Poland. He completed his MSc in 2014 at the Warsaw University of Technology. Subsequently, he received his PhD from the Institute of Organic Chemistry of the Polish Academy of Sciences under the guidance of Prof. D. T. Gryko. His research interests focus on the synthesis of functional aromatic molecules, notably dipyrrolonaphthyridinediones*.

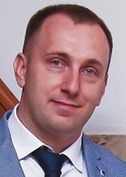

## Biographical Information

*Holger Butenschön obtained his PhD with A. de Meijere in 1983. After a postdoctoral year with Prof. K. P. C. Vollhardt at the University of California at Berkeley, he carried out independent research at the Max‐Planck‐Institute for Coal Research in Mülheim an der Ruhr, Germany. After habilitation in organic chemistry at the University of Hamburg in 1991, he was a Heisenberg fellow (DFG) at the University of Wuppertal. Since 1993 he has been a professor of organic chemistry at Leibniz University Hannover and was a visiting professor at Kyushu University in 1999 as well as at the University of California at Berkeley in 2008*.

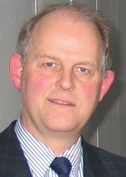

## Biographical Information

*Daniel T. Gryko obtained his PhD from the Institute of Organic Chemistry of the Polish Academy of Sciences in 1997, under the supervision of Prof. J. Jurczak. After postdoctoral research with Prof. J. Lindsey at North Carolina State University, he started his independent career in Poland. Recently, he became Director of the Institute of Organic Chemistry of the Polish Academy of Sciences. He received the Society of Porphyrins and Phthalocyanines Young Investigator Award in 2008 and Foundation for Polish Science Award in 2017*.

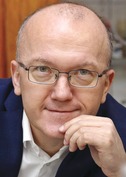

## Supporting information

As a service to our authors and readers, this journal provides supporting information supplied by the authors. Such materials are peer reviewed and may be re‐organized for online delivery, but are not copy‐edited or typeset. Technical support issues arising from supporting information (other than missing files) should be addressed to the authors.

Supplementary

## References

[1] V. Griefsmayer , Justus Liebigs Ann. Chem. 1871, 160, 40–56.

[2] R. Scholl , J. Mansfeld , Ber. Dtsch. Chem. Ges. 1910, 43, 1734–1746.

[3] M. Grzybowski , K. Skonieczny , H. Butenschön , D. T. Gryko , Angew. Chem. Int. Ed. 2013, 52, 9900–9930; 10.1002/anie.201210238 23852649

[4] H. Ito , Y. Segawa , K. Murakami , K. Itami , J. Am. Chem. Soc. 2019, 141, 3–10. 30395456 10.1021/jacs.8b09232

[5] A. Narita , X. Y. Wang , X. Feng , K. Müllen , Chem. Soc. Rev. 2015, 44, 6616–6643. 26186682 10.1039/c5cs00183h

[6] M. Stępień , E. Gońka , M. Żyła , N. Sprutta , Chem. Rev. 2017, 117, 3479–3716. 27258218 10.1021/acs.chemrev.6b00076

[7] F. B. Mallory , C. W. Mallory , in Org. React., Wiley, Hoboken, 1984, pp. 1–456.

[8] K. B. Jørgensen , Molecules 2010, 15, 4334–4358.20657445

[9] A. A. O. Sarhan , C. Bolm , Chem. Soc. Rev. 2009, 38, 2730–2744. 19690750 10.1039/b906026j

[10] K. Morioku , N. Morimoto , Y. Takeuchi , Y. Nishina , Sci. Rep. 2016, 6, 1–8. 27181191 10.1038/srep25824 PMC4867571

[11] A. K. Budniak , M. Masny , K. Prezelj , M. Grzeszkiewicz , J. Gawraczyński , Ł. Dobrzycki , M. K. Cyrański , W. Koźmiński , Z. Mazej , K. J. Fijałkowski , et al., New J. Chem. 2017, 41, 10742–10749.

[12] S. R. Waldvogel , S. Trosien , Chem. Commun. 2012, 48, 9109–9119. 10.1039/c2cc33925k 22822487

[13] S. B. Beil , I. Uecker , P. Franzmann , T. Müller , S. R. Waldvogel , Org. Lett. 2018, 20, 4107–4110. 29920111 10.1021/acs.orglett.8b01664

[14] P. Kovacic , R. M. Lange , J. Org. Chem. 1965, 30, 4251–4254.

[15] B. Kramer , R. Fröhlich , S. R. Waldvogel , Eur. J. Org. Chem. 2003, 3549–3554.

[16] M. Schubert , J. Leppin , K. Wehming , D. Schollmeyer , K. Heinze , S. R. Waldvogel , Angew. Chem. Int. Ed. 2014, 53, 2494–2497; 10.1002/anie.201309287 24478061

[17] S. B. Beil , T. Müller , S. B. Sillart , P. Franzmann , A. Bomm , M. Holtkamp , U. Karst , W. Schade , S. R. Waldvogel , Angew. Chem. Int. Ed. 2018, 57, 2450–2454; 10.1002/anie.201712718 29318724

[18] E. M. Boyd , J. Sperry , Org. Lett. 2015, 17, 1344–1346. 25699647 10.1021/acs.orglett.5b00300

[19] D. R. Armstrong , R. J. Breckenridge , C. Cameron , D. C. Nonhebel , P. L. Pauson , P. G. Perkins , Tetrahedron Lett. 1983, 24, 1071–1074.

[20] Y. E. Lee , T. Cao , C. Torruellas , M. C. Kozlowski , J. Am. Chem. Soc. 2014, 136, 6782–6785. 24797179 10.1021/ja500183z PMC4227839

[21] X. Wu , T. Iwata , A. Scharf , T. Qin , K. D. Reichl , J. A. Porco , J. Am. Chem. Soc. 2018, 140, 5969–5975. 29658717 10.1021/jacs.8b02535 PMC5943148

[22] K. Nakanishi , T. Sasamori , K. Kuramochi , N. Tokitoh , T. Kawabata , K. Tsubaki , J. Org. Chem. 2014, 79, 2625–2631. 24564354 10.1021/jo500085a

[23] K. Hassan , K. Yamashita , K. Hirabayashi , T. Shimizu , K. Nakabayashi , Y. Imai , T. Matsumoto , A. Yamano , K. Sugiura , Chem. Lett. 2015, 44, 1607–1609.

[24] S. Rajbangshi , K. I. Sugiura , Synthesis 2017, 49, 3145–3148.

[25] R. Nazir , A. J. Stasyuk , D. T. Gryko , J. Org. Chem. 2016, 81, 11104–11114. 27788333 10.1021/acs.joc.6b02094

[26] Y. Li , Y. Hong , J. Guo , X. Huang , H. Wei , J. Zhou , T. Qiu , J. Wu , Z. Zeng , Org. Lett. 2017, 19, 5094–5097. 28901146 10.1021/acs.orglett.7b02370

[27] S. Maddala , S. Mallick , P. Venkatakrishnan , J. Org. Chem. 2017, 82, 8958–8972. 28816448 10.1021/acs.joc.7b01377

[28] K. Morimoto , D. Koseki , T. Dohi , Y. Kita , Synlett 2017, 28, 2941–2945.

[29] K. Matsumoto , K. Dougomori , S. Tachikawa , T. Ishii , M. Shindo , Org. Lett. 2014, 16, 4754–4757. 25238149 10.1021/ol502197p

[30] H. Forkosh , V. Vershinin , H. Reiss , D. Pappo , Org. Lett. 2018, 20, 2459–2463. 29608314 10.1021/acs.orglett.8b00800

[31] K. Matsumoto , M. Yoshida , M. Shindo , Angew. Chem. Int. Ed. 2016, 55, 5272–5276; 10.1002/anie.201600400 26996772

[32] R. F. Fritsche , G. Theumer , O. Kataeva , H. J. Knölker , Angew. Chem. Int. Ed. 2017, 56, 549–553; 10.1002/anie.201610168 27918127

[33] S. Mallick , S. Maddala , K. Kollimalayan , P. Venkatakrishnan , J. Org. Chem. 2019, 84, 73–93. 30521754 10.1021/acs.joc.8b02322

[34] J. Yuan , L. Jin , R. Chen , X. Tang , X. Xie , Y. Tang , W. Huang , New J. Chem. 2018, 42, 14704–14708.

[35] M. Müller , C. Kübel , K. Müllen , Chem. Eur. J. 1998, 4, 2099–2109.

[36] J. Wu , W. Pisula , K. Müllen , Chem. Rev. 2007, 107, 718–747. 17291049 10.1021/cr068010r

[37] R. V. Salvatierra , V. H. R. Souza , C. F. Matos , M. M. Oliveira , A. J. G. Zarbin , Carbon 2015, 93, 924–932.

[38] T. Wirtanen , M. K. Mäkelä , J. Sarfraz , P. Ihalainen , S. Hietala , M. Melchionna , J. Helaja , Adv. Synth. Catal. 2015, 357, 3718–3726.

[39] T. Wirtanen , M. Muuronen , J. Hurmalainen , H. M. Tuononen , M. Nieger , J. Helaja , Org. Chem. Front. 2016, 3, 1738–1745.

[40] L. Bering , F. M. Paulussen , A. P. Antonchick , Org. Lett. 2018, 20, 1978–1981. 29547297 10.1021/acs.orglett.8b00521

[41] D. Seidel , V. Lynch , J. L. Sessler , Angew. Chem. Int. Ed. 2002, 41, 1422–1425; 10.1002/1521-3773(20020415)41:8<1422::aid-anie1422>3.0.co;2-o 19750786

[42] T. Köhler , D. Seidel , V. Lynch , F. O. Arp , Z. Ou , K. M. Kadish , J. L. Sessler , J. Am. Chem. Soc. 2003, 125, 6872–6873. 12783532 10.1021/ja035089y

[43] S. C. Gadekar , B. K. Reddy , V. G. Anand , Angew. Chem. Int. Ed. 2013, 52, 7164–7167; 10.1002/anie.201303184 23740888

[44] S. C. Gadekar , B. K. Reddy , S. P. Panchal , V. G. Anand , Chem. Commun. 2016, 52, 4565–4568. 10.1039/c5cc10356h 26939925

[45] K. Zhang , M. Savage , X. Li , Y. Jiang , M. Ishida , K. Mitsuno , S. Karasawa , T. Kato , W. Zhu , S. Yang , et al., Chem. Commun. 2016, 52, 5148–5151. 10.1039/c6cc00707d 26996893

[46] A. Libman , H. Shalit , Y. Vainer , S. Narute , S. Kozuch , D. Pappo , J. Am. Chem. Soc. 2015, 137, 11453–11460. 26287435 10.1021/jacs.5b06494

[47] M. C. Kozlowski , Acc. Chem. Res. 2017, 50, 638–643. 28945389 10.1021/acs.accounts.6b00637 PMC5645065

[48] A. Kirste , G. Schnakenburg , F. Stecker , A. Fischer , S. R. Waldvogel , Angew. Chem. Int. Ed. 2010, 49, 971–975; 10.1002/anie.200904763 20029859

[49] B. Elsler , D. Schollmeyer , K. M. Dyballa , R. Franke , S. R. Waldvogel , Angew. Chem. Int. Ed. 2014, 53, 5210–5213; 10.1002/anie.201400627 24644088

[50] A. Wiebe , D. Schollmeyer , K. M. Dyballa , R. Franke , S. R. Waldvogel , Angew. Chem. Int. Ed. 2016, 55, 11801–11805; 10.1002/anie.201604321 27401116

[51] S. R. Waldvogel , S. Lips , ChemElectroChem 2018, 6, 1649–1660.

[52] S. Möhle , M. Zirbes , E. Rodrigo , T. Gieshoff , A. Wiebe , S. R. Waldvogel , Angew. Chem. Int. Ed. 2018, 57, 6018–6041; 10.1002/anie.201712732 PMC6001547 29359378

[53] A. Kirste , B. Elsler , G. Schnakenburg , S. R. Waldvogel , J. Am. Chem. Soc. 2012, 134, 3571–3576. 22242769 10.1021/ja211005g

[54] B. Elsler , A. Wiebe , D. Schollmeyer , K. M. Dyballa , R. Franke , S. R. Waldvogel , Chem. Eur. J. 2015, 21, 12321–12325. 26189655 10.1002/chem.201501604

[55] L. Schulz , M. Enders , B. Elsler , D. Schollmeyer , K. M. Dyballa , R. Franke , S. R. Waldvogel , Angew. Chem. Int. Ed. 2017, 56, 4877–4881; 10.1002/anie.201612613 28252240

[56] A. Wiebe , S. Lips , D. Schollmeyer , R. Franke , S. R. Waldvogel , Angew. Chem. Int. Ed. 2017, 56, 14727–14731; 10.1002/anie.201708946 28967700

[57] S. Lips , D. Schollmeyer , R. Franke , S. R. Waldvogel , Angew. Chem. Int. Ed. 2018, 57, 13325–13329; 10.1002/anie.201808555 30101511

[58] K. Morimoto , T. Dohi , Y. Kita , Synlett 2017, 28, 1680–1694.

[59] K. Morimoto , K. Sakamoto , Y. Ohnishi , T. Miyamoto , M. Ito , T. Dohi , Y. Kita , Chem. Eur. J. 2013, 19, 8726–8731. 23696108 10.1002/chem.201301028

[60] M. Ito , H. Kubo , I. Itani , K. Morimoto , T. Dohi , Y. Kita , J. Am. Chem. Soc. 2013, 135, 14078–14081. 24028674 10.1021/ja407944p

[61] K. Morimoto , K. Sakamoto , T. Ohshika , T. Dohi , Y. Kita , Angew. Chem. Int. Ed. 2016, 55, 3652–3656; 10.1002/anie.201511007 26879796

[62] N. Y. More , M. Jeganmohan , Org. Lett. 2015, 17, 3042–3045. 26023816 10.1021/acs.orglett.5b01324

[63] E. Gaster , Y. Vainer , A. Regev , S. Narute , K. Sudheendran , A. Werbeloff , H. Shalit , D. Pappo , Angew. Chem. Int. Ed. 2015, 54, 4198–4202; 10.1002/anie.201409694 25655277

[64] H. Shalit , A. Libman , D. Pappo , J. Am. Chem. Soc. 2017, 139, 13404–13413. 28862442 10.1021/jacs.7b05898

[65] H. Reiss , H. Shalit , V. Vershinn , N. Y. More , H. Forckosh , D. Pappo , J. Org. Chem. 2019, 84, 7950–7960. 31064184 10.1021/acs.joc.9b00822

[66] Y. Nieves-Quinones , T. J. Paniak , Y. E. Lee , S. M. Kim , S. Tcyrulnikov , M. C. Kozlowski , J. Am. Chem. Soc. 2019, 141, 10016–10032. 31125210 10.1021/jacs.9b03890 PMC6628261

[67] A. Dyadyuk , K. Sudheendran , Y. Vainer , V. Vershinin , A. I. Shames , D. Pappo , Org. Lett. 2016, 18, 4324–4327. 27529128 10.1021/acs.orglett.6b02064

[68] Y. Sawama , M. Masuda , R. Nakatani , H. Yokoyama , Y. Monguchi , T. Dohi , Y. Kita , H. Sajiki , Adv. Synth. Catal. 2016, 358, 3683–3687.

[69] Y. Tamura , T. Yakura , J. ichi Haruta , Y. Kita , J. Org. Chem. 1987, 52, 3927–3930.

[70] A. S. Mitchell , R. A. Russell , Tetrahedron 1997, 53, 4387–4410.

[71] D. W. Norman , C. A. Carraz , D. J. Hyett , P. G. Pringle , J. B. Sweeney , A. G. Orpen , H. Phetmung , R. L. Wingad , J. Am. Chem. Soc. 2008, 130, 6840–6847. 18454526 10.1021/ja800858x

[72] J. Aydin , K. S. Kumar , M. J. Sayah , O. A. Wallner , K. J. Szabó , J. Org. Chem. 2007, 72, 4689–4697. 17523662 10.1021/jo070288b

[73] T. Wang , H. Ma , S. Zhang , Z. J. Li , M. Zhang , F. Li , F. Sun , J. Xiang , M. Ke , Q. Wang , Org. Lett. 2018, 20, 3591–3595. 29851491 10.1021/acs.orglett.8b01380

[74] K. Morimoto , A. Nakamura , T. Dohi , Y. Kita , Eur. J. Org. Chem. 2016, 4294–4297.

[75] G. Bringmann , R. Walter , R. Weirich , Angew. Chem. Int. Ed. Engl. 1990, 29, 977–991;

[76] M. C. Kozlowski , B. J. Morgan , E. C. Linton , Chem. Soc. Rev. 2009, 38, 3193–3207. 19847351 10.1039/b821092f PMC2904069

[77] G. Bringmann , T. Gulder , T. A. M. Gulder , M. Breuning , Chem. Rev. 2011, 111, 563–639. 20939606 10.1021/cr100155e

[78] J. Wencel-Delord , A. Panossian , F. R. Leroux , F. Colobert , Chem. Soc. Rev. 2015, 44, 3418–3430. 25904287 10.1039/c5cs00012b

[79] J. M. Brunel , Chem. Rev. 2005, 105, 857–898. 15755079 10.1021/cr040079g

[80] M. Nakajima , I. Miyoshi , K. Kanayama , S. I. Hashimoto , M. Noji , K. Koga , J. Org. Chem. 1999, 64, 2264–2271.

[81] X. Li , J. Yang , M. C. Kozlowski , Org. Lett. 2001, 3, 1137–1140. 11348178 10.1021/ol015595x

[82] K. H. Kim , D. W. Lee , Y. S. Lee , D. H. Ko , D. C. Ha , Tetrahedron 2004, 60, 9037–9042.

[83] S. E. Allen , R. R. Walvoord , R. Padilla-Salinas , M. C. Kozlowski , Chem. Rev. 2013, 113, 6234–6458. 23786461 10.1021/cr300527g PMC3818381

[84] N. B. Barhate , C. T. Chen , Org. Lett. 2002, 4, 2529–2532. 12123368 10.1021/ol026156g

[85] C. Y. Chu , B. J. Uang , Tetrahedron: Asymmetry 2003, 14, 53–55.

[86] Q. X. Guo , Z. J. Wu , Z. Bin Luo , Q. Z. Liu , J. L. Ye , S. W. Luo , L. F. Cun , L. Z. Gong , J. Am. Chem. Soc. 2007, 129, 13927–13938. 17956093 10.1021/ja074322f

[87] S. Takizawa , T. Katayama , H. Somei , Y. Asano , T. Yoshida , C. Kameyama , D. Rajesh , K. Onitsuka , T. Suzuki , M. Mikami , et al., Tetrahedron 2008, 64, 3361–3371.

[88] S. Takizawa , H. Gröger , H. Sasai , Chem. Eur. J. 2015, 21, 8992–8997. 25809123 10.1002/chem.201406444

[89] H. Egami , T. Katsuki , J. Am. Chem. Soc. 2009, 131, 6082–6083. 19361160 10.1021/ja901391u

[90] H. Shalit , A. Dyadyuk , D. Pappo , J. Org. Chem. 2019, 84, 1677–1686. 30657316 10.1021/acs.joc.8b03084

[91] H. Kang , M. R. Herling , K. A. Niederer , Y. E. Lee , P. Vasu Govardhana Reddy , S. Dey , S. E. Allen , P. Sung , K. Hewitt , C. Torruellas , et al., J. Org. Chem. 2018, 83, 14362–14384. 30376626 10.1021/acs.joc.8b02083 PMC6467780

[92] S. Narute , R. Parnes , F. D. Toste , D. Pappo , J. Am. Chem. Soc. 2016, 138, 16553–16560. 27959518 10.1021/jacs.6b11198

[93] M. Sako , S. Takizawa , Y. Yoshida , H. Sasai , Tetrahedron: Asymmetry 2015, 26, 613–616.

[94] S. Takizawa , J. Kodera , Y. Yoshida , M. Sako , S. Breukers , D. Enders , H. Sasai , Tetrahedron 2014, 70, 1786–1793.

[95] H. Y. Kim , S. Takizawa , H. Sasai , K. Oh , Org. Lett. 2017, 19, 3867–3870. 28696128 10.1021/acs.orglett.7b01734

[96] M. Sako , Y. Takeuchi , T. Tsujihara , J. Kodera , T. Kawano , S. Takizawa , H. Sasai , J. Am. Chem. Soc. 2016, 138, 11481–11484. 27574874 10.1021/jacs.6b07424

[97] H. Kang , Y. E. Lee , P. V. G. Reddy , S. Dey , S. E. Allen , K. A. Niederer , P. Sung , K. Hewitt , C. Torruellas , M. R. Herling , et al., Org. Lett. 2017, 19, 5505–5508. 29022352 10.1021/acs.orglett.7b02552 PMC5654492

[98] H. Kang , C. Torruellas , J. Liu , M. C. Kozlowski , Org. Lett. 2018, 20, 5554–5558. 30207731 10.1021/acs.orglett.8b02183 PMC6174078

[99] M. Sako , T. Aoki , N. Zumbrägel , L. Schober , H. Gröger , S. Takizawa , H. Sasai , J. Org. Chem. 2019, 84, 1580–1587. 30501179 10.1021/acs.joc.8b02494

[100] Y. Wu , J. Wang , F. Mao , F. Y. Kwong , Chem. Asian J. 2014, 9, 26–47. 24123795 10.1002/asia.201300990

[101] I. Hussain , T. Singh , Adv. Synth. Catal. 2014, 356, 1661–1696.

[102] C. Liu , J. Yuan , M. Gao , S. Tang , W. Li , R. Shi , A. Lei , Chem. Rev. 2015, 115, 12138–12204. 26558751 10.1021/cr500431s

[103] Y. Yang , J. Lan , J. You , Chem. Rev. 2017, 117, 8787–8863. 28085272 10.1021/acs.chemrev.6b00567

[104] Y. F. Zhang , Z. J. Shi , Acc. Chem. Res. 2019, 52, 161–169. 30376296 10.1021/acs.accounts.8b00408

[105] L. H. Zou , J. Mottweiler , D. L. Priebbenow , J. Wang , J. A. Stubenrauch , C. Bolm , Chem. Eur. J. 2013, 19, 3302–3305. 23401050 10.1002/chem.201204502

[106] R. Odani , K. Hirano , T. Satoh , M. Miura , J. Org. Chem. 2013, 78, 11045–11052. 24102696 10.1021/jo402078q

[107] X. Qin , H. Liu , D. Qin , Q. Wu , J. You , D. Zhao , Q. Guo , X. Huang , J. Lan , Chem. Sci. 2013, 4, 1964–1969.

[108] V. P. Reddy , R. Qiu , T. Iwasaki , N. Kambe , Org. Lett. 2013, 15, 1290–1293. 23441905 10.1021/ol400230y

[109] Q. Wu , Y. Chen , D. Yan , M. Zhang , Y. Lu , W. Y. Sun , J. Zhao , Chem. Sci. 2017, 8, 169–173. 28451162 10.1039/c6sc03169b PMC5308286

[110] J. Dong , Z. Long , F. Song , N. Wu , Q. Guo , J. Lan , J. You , Angew. Chem. Int. Ed. 2013, 52, 580–584; 10.1002/anie.201207196 23184621

[111] D. Qin , J. Wang , X. Qin , C. Wang , G. Gao , J. You , Chem. Commun. 2015, 51, 6190–6193. 10.1039/c5cc00387c 25753941

[112] Y. Huang , D. Wu , J. Huang , Q. Guo , J. Li , J. You , Angew. Chem. Int. Ed. 2014, 53, 12158–12162; 10.1002/anie.201406445 25219784

[113] H. Gong , H. Zeng , F. Zhou , C. J. Li , Angew. Chem. Int. Ed. 2015, 54, 5718–5721; 10.1002/anie.201500220 25765625

[114] Y. Liu , Y. Zhu , C. Li , Front. Chem. Sci. Eng. 2018, 12, 3–8.

[115] H. Zhang , G. J. Deng , S. Li , C. J. Li , H. Gong , RSC Adv. 2016, 6, 91617–91620.

[116] F. Yang , F. Song , W. Li , J. Lan , J. You , RSC Adv. 2013, 3, 9649–9652.

[117] L. Y. Jiao , P. Smirnov , M. Oestreich , Org. Lett. 2014, 16, 6020–6023. 25375317 10.1021/ol503035z

[118] Z. Yang , F. C. Qiu , J. Gao , Z. W. Li , B. T. Guan , Org. Lett. 2015, 17, 4316–4319. 26308790 10.1021/acs.orglett.5b02135

[119] C. Zhang , Y. Rao , Org. Lett. 2015, 17, 4456–4459. 26322534 10.1021/acs.orglett.5b02115

[120] S. J. Lou , Y. J. Mao , D. Q. Xu , J. Q. He , Q. Chen , Z. Y. Xu , ACS Catal. 2016, 6, 3890–3894.

[121] G. L. Gao , W. Xia , P. Jain , J. Q. Yu , Org. Lett. 2016, 18, 744–747. 26835845 10.1021/acs.orglett.5b03712 PMC4821414

[122] M. Wehmeier , R. Epsch , J. D. Brand , C. Kübel , K. Müllen , J. P. Rabe , S. Ito , Chem. Eur. J. 2000, 6, 4327–4342. 11140962 10.1002/1521-3765(20001201)6:23<4327::aid-chem4327>3.0.co;2-7

[123] A. Fechtenkötter , K. Saalwächter , M. A. Harbison , K. Müllen , H. W. Spiess , Angew. Chem. Int. Ed. 1999, 38, 3039–3042; 10540414

[124] R. Dorel , C. Manzano , M. Grisolia , W. H. Soe , C. Joachim , A. M. Echavarren , Chem. Commun. 2015, 51, 6932–6935. 10.1039/c5cc00693g 25794244

[125] H. Arslan , F. J. Uribe-Romo , B. J. Smith , W. R. Dichtel , Chem. Sci. 2013, 4, 3973–3978.

[126] T. L. Wu , C. H. Kuo , B. C. Lin , Y. T. Tao , C. P. Hsu , R. S. Liu , J. Mater. Chem. C 2015, 3, 7583–7588.

[127] J. Liu , B. W. Li , Y. Z. Tan , A. Giannakopoulos , C. Sanchez-Sanchez , D. Beljonne , P. Ruffieux , R. Fasel , X. Feng , K. Müllen , J. Am. Chem. Soc. 2015, 137, 6097–6103. 25909566 10.1021/jacs.5b03017 PMC4456008

[128] T. S. Navale , M. V. Ivanov , M. M. Hossain , R. Rathore , Angew. Chem. Int. Ed. 2018, 57, 790–794; 10.1002/anie.201711739 29194947

[129] G. Li , K. Y. Yoon , X. Zhong , X. Zhu , G. Dong , Chem. Eur. J. 2016, 22, 9116–9120. 27159538 10.1002/chem.201602007

[130] Y. Koga , T. Kaneda , Y. Saito , K. Murakami , K. Itami , Science 2018, 359, 435–439. 29371465 10.1126/science.aap9801

[131] C. P. Sen , S. Valiyaveettil , Chem. Eur. J. 2017, 23, 1686–1693. 27897361 10.1002/chem.201604778

[132] C. Zhu , D. Wang , D. Wang , Y. Zhao , W. Y. Sun , Z. Shi , Angew. Chem. Int. Ed. 2018, 57, 8848–8853; 10.1002/anie.201803603 29663643

[133] S. Kumar , M. T. Ho , Y. T. Tao , Org. Lett. 2016, 18, 200–203. 26714237 10.1021/acs.orglett.5b03291

[134] Y. T. Tao , S. Pola , S. Kumar , M. M. Islam , J. Org. Chem. 2017, 82, 8067–8071. 28697601 10.1021/acs.joc.7b01328

[135] S. Kumar , D. C. Huang , S. Venkateswarlu , Y. T. Tao , J. Org. Chem. 2018, 83, 11614–11622. 30141326 10.1021/acs.joc.8b01582

[136] S. Kumar , Y. T. Tao , Org. Lett. 2018, 20, 2320–2323. 29620904 10.1021/acs.orglett.8b00666

[137] Z. A. Kasun , H. Sato , J. Nie , Y. Mori , J. A. Bender , S. T. Roberts , M. J. Krische , Chem. Sci. 2018, 9, 7866–7873. 30429996 10.1039/c8sc03236j PMC6194800

[138] Y. Chen , T. Marszalek , T. Fritz , M. Baumgarten , M. Wagner , W. Pisula , L. Chen , K. Müllen , Chem. Commun. 2017, 53, 8474–8477. 10.1039/c7cc03709k 28703255

[139] K. Baumgärtner , A. L. Meza Chincha , A. Dreuw , F. Rominger , M. Mastalerz , Angew. Chem. Int. Ed. 2016, 55, 15594–15598; 10.1002/anie.201607740 27649907

[140] K. Q. Zhao , M. Jing , L. L. An , J. Q. Du , Y. H. Wang , P. Hu , B. Q. Wang , H. Monobe , B. Heinrich , B. Donnio , J. Mater. Chem. C 2017, 5, 669–682.

[141] Y. Tokoro , A. Oishi , S.-ichi Fukuzawa , Chem. Eur. J. 2016, 22, 13908–13915. 27515137 10.1002/chem.201602474

[142] A. Naibi Lakshminarayana , J. Chang , J. Luo , B. Zheng , K. W. Huang , C. Chi , Chem. Commun. 2015, 51, 3604–3607. 10.1039/c4cc09812a 25634022

[143] M. Kawamura , E. Tsurumaki , S. Toyota , Synthesis 2018, 50, 134–138.

[144] L. J. Purvis , X. Gu , S. Ghosh , Z. Zhang , C. J. Cramer , C. J. Douglas , J. Org. Chem. 2018, 83, 1828–1841. 29357664 10.1021/acs.joc.7b02756

[145] D. Lungerich , J. F. Hitzenberger , W. Donaubauer , T. Drewello , N. Jux , Chem. Eur. J. 2016, 22, 16697. 10.1002/chem.201603789 27661059

[146] W. Zeng , H. Phan , T. S. Herng , T. Y. Gopalakrishna , N. Aratani , Z. Zeng , H. Yamada , J. Ding , J. Wu , Chem 2017, 2, 81–92.

[147] T. Hensel , N. N. Andersen , M. Plesner , M. Pittelkow , Synlett 2016, 27, 498–525.

[148] Q. Zhang , H. Peng , G. Zhang , Q. Lu , J. Chang , Y. Dong , X. Shi , J. Wei , J. Am. Chem. Soc. 2014, 136, 5057–5064. 24564649 10.1021/ja413018f

[149] X. Zhang , Z. Xu , W. Si , K. Oniwa , M. Bao , Y. Yamamoto , T. Jin , Nat. Commun. 2017, 8, 15073. 28440319 10.1038/ncomms15073 PMC5414065

[150] S. Nobusue , K. Fujita , Y. Tobe , Org. Lett. 2017, 19, 3227–3230. 28585441 10.1021/acs.orglett.7b01341

[151] J. Liu , A. Narita , S. Osella , W. Zhang , D. Schollmeyer , D. Beljonne , X. Feng , K. Müllen , J. Am. Chem. Soc. 2016, 138, 2602–2608. 26859522 10.1021/jacs.5b10399

[152] J. Liu , S. Osella , J. Ma , R. Berger , D. Beljonne , D. Schollmeyer , X. Feng , K. Müllen , J. Am. Chem. Soc. 2016, 138, 8364–8367. 27355697 10.1021/jacs.6b04426

[153] J. He , S. Mathew , Z. J. Kinney , R. M. Warrell , J. S. Molina , C. S. Hartley , Chem. Commun. 2015, 51, 7245–7248. 10.1039/c5cc00826c 25814021

[154] Chaolumen , M. Murata , A. Wakamiya , Y. Murata , Angew. Chem. Int. Ed. 2017, 56, 5082–5086; 10.1002/anie.201701054 28370944

[155] M. Shekhirev , A. Sinitskii , Phys. Sci. Rev. 2017, 2, 1–21.

[156] A. Narita , Z. Chen , Q. Chen , K. Müllen , Chem. Sci. 2019, 10, 964–975. 30774890 10.1039/c8sc03780a PMC6349060

[157] A. Narita , X. Feng , Y. Hernandez , S. A. Jensen , M. Bonn , H. Yang , I. A. Verzhbitskiy , C. Casiraghi , M. R. Hansen , A. H. R. Koch , et al., Nat. Chem. 2014, 6, 126–132. 24451588 10.1038/nchem.1819

[158] M. G. Schwab , A. Narita , S. Osella , Y. Hu , A. Maghsoumi , A. Mavrinsky , W. Pisula , C. Castiglioni , M. Tommasini , D. Beljonne , et al., Chem. Asian J. 2015, 10, 2134–2138. 26062724 10.1002/asia.201500450

[159] I. C. Y. Hou , Y. Hu , A. Narita , K. Müllen , Polym. J. 2018, 50, 3–20.

[160] S. Grätz , D. Beyer , V. Tkachova , S. Hellmann , R. Berger , X. Feng , L. Borchardt , Chem. Commun. 2018, 54, 5307–5310. 10.1039/c8cc01993b 29651492

[161] M. Tasior , D. Kim , S. Singha , M. Krzeszewski , K. H. Ahn , D. T. Gryko , J. Mater. Chem. C 2015, 3, 1421–1446.

[162] M. K. Węcławski , M. Tasior , T. Hammann , P. J. Cywiński , D. T. Gryko , Chem. Commun. 2014, 50, 9105–9108. 10.1039/c4cc03078h 24985198

[163] M. K. Węcławski , I. Deperasińska , M. Banasiewicz , D. C. Young , A. Leniak , D. T. Gryko , Chem. Asian J. 2019, 14, 1763–1770. 30022613 10.1002/asia.201800757

[164] M. Krzeszewski , D. Gryko , D. T. Gryko , Acc. Chem. Res. 2017, 50, 2334–2345. 28795799 10.1021/acs.accounts.7b00275

[165] M. Krzeszewski , D. T. Gryko , J. Org. Chem. 2015, 80, 2893–2899. 25692662 10.1021/acs.joc.5b00052

[166] A. J. Stone , D. J. Wales , Chem. Phys. Lett. 1986, 128, 501–503.

[167] M. T. Lusk , D. T. Wu , L. D. Carr , Phys. Rev. B 2010, 81, 155444.

[168] M. Krzeszewski , T. Kodama , E. M. Espinoza , V. I. Vullev , T. Kubo , D. T. Gryko , Chem. Eur. J. 2016, 22, 16478–16488. 27659591 10.1002/chem.201603282

[169] S. Mishra , M. Krzeszewski , C. A. Pignedoli , P. Ruffieux , R. Fasel , D. T. Gryko , Nat. Commun. 2018, 9, 1714. 29712921 10.1038/s41467-018-04144-5 PMC5928119

[170] M. Krzeszewski , K. Sahara , Y. M. Poronik , T. Kubo , D. T. Gryko , Org. Lett. 2018, 20, 1517–1520. 29513012 10.1021/acs.orglett.8b00223

[171] M. Krzeszewski , P. Świder , Ł. Dobrzycki , M. K. Cyrański , W. Danikiewicz , D. T. Gryko , Chem. Commun. 2016, 52, 11539–11542. 10.1039/c6cc05904j 27709209

[172] T. Yang , J. Pu , J. Zhang , W. Wang , J. Org. Chem. 2013, 78, 4857–4866. 23600443 10.1021/jo400425n

[173] H. Zhong , C. H. Wu , C. Z. Li , J. Carpenter , C. C. Chueh , J. Y. Chen , H. Ade , A. K. Y. Jen , Adv. Mater. 2016, 28, 951–958. 26638861 10.1002/adma.201504120

[174] P. E. Hartnett , H. S. S. Ramakrishna Matte , N. D. Eastham , N. E. Jackson , Y. Wu , L. X. Chen , M. A. Ratner , R. P. H. Chang , M. C. Hersam , M. R. Wasielewski , et al., Chem. Sci. 2016, 7, 3543–3555. 29997846 10.1039/c5sc04956c PMC6007210

[175] C. Yu , L. Jiao , T. Li , Q. Wu , W. Miao , J. Wang , Y. Wei , X. Mu , E. Hao , Chem. Commun. 2015, 51, 16852–16855. 10.1039/c5cc07304a 26437694

[176] E. Heyer , P. Retailleau , R. Ziessel , Org. Lett. 2014, 16, 2330–2333. 24720621 10.1021/ol500572t

[177] W. Sheng , Y. Q. Zheng , Q. Wu , Y. Wu , C. Yu , L. Jiao , E. Hao , J. Y. Wang , J. Pei , Org. Lett. 2017, 19, 2893–2896. 28530398 10.1021/acs.orglett.7b01133

[178] J. Cui , W. Sheng , Q. Wu , C. Yu , E. Hao , P. Bobadova-Parvanova , M. Storer , A. M. Asiri , H. M. Marwani , L. Jiao , Chem. Asian J. 2017, 12, 2486–2493. 28730703 10.1002/asia.201700876

[179] W. Sheng , Y. Wu , C. Yu , P. Bobadova-Parvanova , E. Hao , L. Jiao , Org. Lett. 2018, 20, 2620–2623. 29667832 10.1021/acs.orglett.8b00820

[180] K. Skonieczny , J. Jaźwiński , D. T. Gryko , Synthesis 2017, 49, 4651–4662.

[181] T. Ono , N. Xu , D. Koga , T. Ideo , M. Sugimoto , Y. Hisaeda , RSC Adv. 2018, 8, 39269–39273. 10.1039/c8ra09040h PMC9090978 35558012

[182] D. Firmansyah , S. J. Hong , R. Dutta , Q. He , J. Bae , H. Jo , H. Kim , K. M. Ok , V. M. Lynch , H. R. Byon , et al., Chem. Eur. J. 2019, 25, 3525–3531. 30684359 10.1002/chem.201900022

[183] M. Takase , V. Enkelmann , D. Sebastiani , M. Baumgarten , K. Müllen , Angew. Chem. Int. Ed. 2007, 46, 5524–5527; 10.1002/anie.200701452 17582806

[184] M. Takase , T. Narita , W. Fujita , M. S. Asano , T. Nishinaga , H. Benten , K. Yoza , K. Müllen , J. Am. Chem. Soc. 2013, 135, 8031–8040. 23662671 10.1021/ja402371f

[185] M. Zyła , E. Gońka , P. J. Chmielewski , J. Cybińska , M. Stępień , Chem. Sci. 2016, 7, 286–294. 29861982 10.1039/c5sc03280f PMC5952525

[186] E. Gońka , P. J. Chmielewski , T. Lis , M. Stępień , J. Am. Chem. Soc. 2014, 136, 16399–16410. 25348930 10.1021/ja508963v

[187] K. Oki , M. Takase , S. Mori , A. Shiotari , Y. Sugimoto , K. Ohara , T. Okujima , H. Uno , J. Am. Chem. Soc. 2018, 140, 10430–10434. 30068084 10.1021/jacs.8b06079

[188] F. Chen , Y. S. Hong , S. Shimizu , D. Kim , T. Tanaka , A. Osuka , Angew. Chem. Int. Ed. 2015, 54, 10639–10642; 10.1002/anie.201505124 26216171

[189] D. Myśliwiec , M. Stępień , Angew. Chem. Int. Ed. 2013, 52, 1713–1717; 10.1002/anie.201208547 23255207

[190] M. Kondratowicz , D. Myśliwiec , T. Lis , M. Stępień , Chem. Eur. J. 2014, 20, 14981–14985. 25303207 10.1002/chem.201404470

[191] F. Chen , T. Tanaka , T. Mori , A. Osuka , Chem. Eur. J. 2018, 24, 7489–7497. 29533480 10.1002/chem.201800617

[192] M. Takase , A. Inabe , Y. Sugawara , W. Fujita , T. Nishinaga , K. Nomura , Org. Lett. 2013, 15, 3202–3205. 23790099 10.1021/ol400882q

[193] L. Ji , S. Griesbeck , T. B. Marder , Chem. Sci. 2017, 8, 846–863. 28572897 10.1039/c6sc04245g PMC5452272

[194] M. Hirai , N. Tanaka , M. Sakai , S. Yamaguchi , Chem. Rev. 2019, 119, 8291–8331. 30860363 10.1021/acs.chemrev.8b00637

[195] S. Saito , K. Matsuo , S. Yamaguchi , J. Am. Chem. Soc. 2012, 134, 9130–9133. 22595007 10.1021/ja3036042

[196] C. Dou , S. Saito , K. Matsuo , I. Hisaki , S. Yamaguchi , Angew. Chem. Int. Ed. 2012, 51, 12206–12210; 10.1002/anie.201206699 23081889

[197] D.-T. Yang , T. Nakamura , Z. He , X. Wang , A. Wakamiya , T. Peng , S. Wang , Org. Lett. 2018, 20, 6741–6745. 30350666 10.1021/acs.orglett.8b02850

[198] J. P. Lewtak , D. T. Gryko , Chem. Commun. 2012, 48, 10069–10086. 10.1039/c2cc31279d 22649792

[199] T. Tanaka , A. Osuka , Chem. Eur. J. 2018, 24, 17188–17200. 29943429 10.1002/chem.201802810

[200] S. Ooi , T. Tanaka , K. H. Park , D. Kim , A. Osuka , Angew. Chem. Int. Ed. 2016, 55, 6535–6539; 10.1002/anie.201601864 27037528

[201] S. Ooi , T. Tanaka , K. H. Park , S. Lee , D. Kim , A. Osuka , Angew. Chem. Int. Ed. 2015, 54, 3107–3111; 10.1002/anie.201411242 25573778

[202] M. A. Majewski , M. Stępień , Angew. Chem. Int. Ed. 2019, 58, 86–116; 10.1002/anie.201807004 30006951

[203] Y. Sakamoto , T. Suzuki , J. Am. Chem. Soc. 2013, 135, 14074–14077. 24015972 10.1021/ja407842z

[204] C. N. Feng , M. Y. Kuo , Y. T. Wu , Angew. Chem. Int. Ed. 2013, 52, 7791–7794; 10.1002/anie.201303875 23794166

[205] K. Y. Cheung , C. K. Chan , Z. Liu , Q. Miao , Angew. Chem. Int. Ed. 2017, 56, 9003–9007; 10.1002/anie.201703754 28471075

[206] R. W. Miller , S. E. Averill , S. J. Van Wyck , A. C. Whalley , J. Org. Chem. 2016, 81, 12001–12005. 27934450 10.1021/acs.joc.6b02244

[207] S. H. Pun , C. K. Chan , J. Luo , Z. Liu , Q. Miao , Angew. Chem. Int. Ed. 2018, 57, 1581–1586; 10.1002/anie.201711437 29251395

[208] K. Y. Cheung , X. Xu , Q. Miao , J. Am. Chem. Soc. 2015, 137, 3910–3914. 25741577 10.1021/jacs.5b00403

[209] J. Luo , X. Xu , R. Mao , Q. Miao , J. Am. Chem. Soc. 2012, 134, 13796–13803. 22827492 10.1021/ja3054354

[210] W. S. Wong , C. F. Ng , D. Kuck , H. F. Chow , Angew. Chem. Int. Ed. 2017, 56, 12356–12360; 10.1002/anie.201707505 28766911

[211] W. S. Wong , H. W. Tse , E. Cheung , D. Kuck , H. F. Chow , J. Org. Chem. 2019, 84, 869–878. 30550282 10.1021/acs.joc.8b02719

[212] K. Kawasumi , Q. Zhang , Y. Segawa , L. T. Scott , K. Itami , Nat. Chem. 2013, 5, 739–744. 23965674 10.1038/nchem.1704

[213] K. Kato , Y. Segawa , L. T. Scott , K. Itami , Chem. Asian J. 2015, 10, 1635–1639. 26062779 10.1002/asia.201500560

[214] H. A. Lin , Y. Sato , Y. Segawa , T. Nishihara , N. Sugimoto , L. T. Scott , T. Higashiyama , K. Itami , Angew. Chem. Int. Ed. 2018, 57, 2874–2878; 10.1002/anie.201713387 29380493

[215] H.-A. Lin , K. Kato , Y. Segawa , L. T. Scott , K. Itami , Chem. Sci. 2019, 10, 2326–2330. 30881659 10.1039/c8sc04470h PMC6385676

[216] T. Amaya , T. Ito , T. Hirao , Angew. Chem. Int. Ed. 2015, 54, 5483–5487; 10.1002/anie.201500548 25757861

[217] E. U. Mughal , D. Kuck , Chem. Commun. 2012, 48, 8880–8882. 10.1039/c2cc34245f 22810270

[218] E. U. Mughal , B. Neumann , H. G. Stammler , D. Kuck , Eur. J. Org. Chem. 2014, 7469–7480.

[219] H. W. Ip , C. F. Ng , H. F. Chow , D. Kuck , J. Am. Chem. Soc. 2016, 138, 13778–13781. 27478989 10.1021/jacs.6b05820

[220] H. W. Ip , H. F. Chow , D. Kuck , Org. Chem. Front. 2017, 4, 817–822.

[221] L. He , C. F. Ng , Y. Li , Z. Liu , D. Kuck , H. F. Chow , Angew. Chem. Int. Ed. 2018, 57, 13635–13639; 10.1002/anie.201808461 30152145

[222] J. N. Smith , N. T. Lucas , Chem. Commun. 2018, 54, 4716–4719. 10.1039/c8cc01903g 29683182

[223] M. S. Newman , D. Lednicer , J. Am. Chem. Soc. 1956, 78, 4765–4770.

[224] Y. Shen , C. F. Chen , Chem. Rev. 2012, 112, 1463–1535. 22017405 10.1021/cr200087r

[225] M. Gingras , Chem. Soc. Rev. 2013, 42, 968–1006. 23151799 10.1039/c2cs35154d

[226] A. Pradhan , P. Dechambenoit , H. Bock , F. Durola , Angew. Chem. Int. Ed. 2011, 50, 12582–12585; 10.1002/anie.201105105 22057825

[227] A. Pradhan , P. Dechambenoit , H. Bock , F. Durola , J. Org. Chem. 2013, 78, 2266–2274. 23374076 10.1021/jo3027752

[228] A. Pradhan , P. Dechambenoit , H. Bock , F. Durola , Chem. Eur. J. 2016, 22, 18227–18235. 27723150 10.1002/chem.201603702

[229] Y. Hu , X.-Y. Wang , P.-X. Peng , X.-C. Wang , X.-Y. Cao , X. Feng , K. Müllen , A. Narita , Angew. Chem. Int. Ed. 2017, 56, 3374–3378; 10.1002/anie.201610434 27966818

[230] R. Yamano , Y. Shibata , K. Tanaka , Chem. Eur. J. 2018, 24, 6364–6370. 29349825 10.1002/chem.201706008

[231] Y. Yang , L. Yuan , B. Shan , Z. Liu , Q. Miao , Chem. Eur. J. 2016, 22, 18620–18627. 27709688 10.1002/chem.201604649

[232] T. Fujikawa , Y. Segawa , K. Itami , J. Am. Chem. Soc. 2015, 137, 7763–7768. 26028308 10.1021/jacs.5b03118

[233] T. Fujikawa , Y. Segawa , K. Itami , J. Am. Chem. Soc. 2016, 138, 3587–3595. 26918641 10.1021/jacs.6b01303

[234] T. Fujikawa , N. Mitoma , A. Wakamiya , A. Saeki , Y. Segawa , K. Itami , Org. Biomol. Chem. 2017, 15, 4697–4703. 28516991 10.1039/c7ob00987a

[235] F. Schlütter , T. Nishiuchi , V. Enkelmann , K. Müllen , Angew. Chem. Int. Ed. 2014, 53, 1538–1542; 10.1002/anie.201309324 24492971

[236] D. Reger , P. Haines , F. W. Heinemann , D. M. Guldi , N. Jux , Angew. Chem. Int. Ed. 2018, 57, 5938–5942; 10.1002/anie.201800585 29508521

[237] C. M. Cruz , S. Castro-Fernández , E. Maçôas , J. M. Cuerva , A. G. Campaña , Angew. Chem. Int. Ed. 2018, 57, 14782–14786; 10.1002/anie.201808178 30144368

[238] I. R. Márquez , N. Fuentes , C. M. Cruz , V. Puente-Muñoz , L. Sotorrios , M. L. Marcos , D. Choquesillo-Lazarte , B. Biel , L. Crovetto , E. Gómez-Bengoa , et al., Chem. Sci. 2017, 8, 1068–1074. 28451246 10.1039/c6sc02895k PMC5357993

[239] C. M. Cruz , I. R. Márquez , I. F. A. Mariz , V. Blanco , C. Sánchez-Sánchez , J. M. Sobrado , J. A. Martín-Gago , J. M. Cuerva , E. Maçôas , A. G. Campaña , Chem. Sci. 2018, 9, 3917–3924. 29780523 10.1039/c8sc00427g PMC5934837

[240] Y. Zhu , Z. Xia , Z. Cai , Z. Yuan , N. Jiang , T. Li , Y. Wang , X. Guo , Z. Li , S. Ma , et al., J. Am. Chem. Soc. 2018, 140, 4222–4226. 29537262 10.1021/jacs.8b01447

[241] Y. Zhu , X. Guo , Y. Li , J. Wang , J. Am. Chem. Soc. 2019, 141, 5511–5517. 30860370 10.1021/jacs.9b01266

[242] Q. Xiao , T. Sakurai , T. Fukino , K. Akaike , Y. Honsho , A. Saeki , S. Seki , K. Kato , M. Takata , T. Aida , J. Am. Chem. Soc. 2013, 135, 18268–18271. 24279901 10.1021/ja4092769

[243] D. Waghray , C. De Vet , K. Karypidou , W. Dehaen , J. Org. Chem. 2013, 78, 11147–11154. 24147631 10.1021/jo401807x

[244] F. Chen , T. Tanaka , Y. S. Hong , T. Mori , D. Kim , A. Osuka , Angew. Chem. Int. Ed. 2017, 56, 14688–14693; 10.1002/anie.201708429 28948686

[245] F. E. Golling , M. Quernheim , M. Wagner , T. Nishiuchi , K. Müllen , Angew. Chem. Int. Ed. 2014, 53, 1525–1528; 10.1002/anie.201309104 24453051

[246] F. E. Golling , S. Osella , M. Quernheim , M. Wagner , D. Beljonne , K. Müllen , Chem. Sci. 2015, 6, 7072–7078. 28757981 10.1039/c5sc02547h PMC5510010

[247] T. J. Sisto , L. N. Zakharov , B. M. White , R. Jasti , Chem. Sci. 2016, 7, 3681–3688. 29997859 10.1039/c5sc04218f PMC6008588

[248] K. Y. Cheung , S. Gui , C. Deng , H. Liang , Z. Xia , Z. Liu , L. Chi , Q. Miao , Chem 2019, 5, 838–847.

[249] Y. Yang , M. Chu , Q. Miao , Org. Lett. 2018, 20, 4259–4262. 29953241 10.1021/acs.orglett.8b01668

[250] G. Otero , G. Biddau , C. Sánchez-Sánchez , R. Caillard , M. F. López , C. Rogero , F. J. Palomares , N. Cabello , M. A. Basanta , J. Ortega , et al., Nature 2008, 454, 865–868. 18704082 10.1038/nature07193

[251] G. Beernink , M. Gunia , F. Dötz , H. Öström , K. Weiss , K. Müllen , C. Wöll , ChemPhysChem 2001, 2, 317–320. 23696505 10.1002/1439-7641(20010518)2:5<317::AID-CPHC317>3.0.CO;2-L

[252] K. T. Rim , M. Siaj , S. Xiao , M. Myers , V. D. Carpentier , L. Liu , C. Su , M. L. Steigerwald , M. S. Hybertsen , P. H. McBreen , et al., Angew. Chem. Int. Ed. 2007, 46, 7891–7895; 10.1002/anie.200701117 17879251

[253] J. R. Sanchez-Valencia , T. Dienel , O. Gröning , I. Shorubalko , A. Mueller , M. Jansen , K. Amsharov , P. Ruffieux , R. Fasel , Nature 2014, 512, 61–64. 25100481 10.1038/nature13607

[254] J. Krüger , F. García , F. Eisenhut , D. Skidin , J. M. Alonso , E. Guitián , D. Pérez , G. Cuniberti , F. Moresco , D. Peña , Angew. Chem. Int. Ed. 2017, 56, 11945–11948; 10.1002/anie.201706156 28771920

[255] C. Rogers , C. Chen , Z. Pedramrazi , A. A. Omrani , H. Z. Tsai , H. S. Jung , S. Lin , M. F. Crommie , F. R. Fischer , Angew. Chem. Int. Ed. 2015, 54, 15143–15146; 10.1002/anie.201507104 26482225

[256] J. Hieulle , E. Carbonell-Sanromà , M. Vilas-Varela , A. Garcia-Lekue , E. Guitián , D. Peña , J. I. Pascual , Nano Lett. 2018, 18, 418–423. 29232951 10.1021/acs.nanolett.7b04309

[257] A. L. Pinardi , J. I. Martínez , A. Jančařík , I. G. Stará , I. Starý , M. F. López , J. Méndez , J. Á. Martín-Gago , Chem. Commun. 2014, 50, 1555–1557. 10.1039/c3cc47399f 24382373

[258] On-Surface Synthesis (Ed.: A. Gourdon), Springer, Cham, 2016.

[259] F. R. Fischer , in Adv. Polym. Sci., Springer, Cham, 2017, pp. 33–65.

[260] L. Talirz , P. Ruffieux , R. Fasel , Adv. Mater. 2016, 28, 6222–6231. 26867990 10.1002/adma.201505738

[261] T. Wang , J. Zhu , Surf. Sci. Rep. 2019, 74, 97–140.

[262] M. Treier , C. A. Pignedoli , T. Laino , R. Rieger , K. Müllen , D. Passerone , R. Fasel , Nat. Chem. 2011, 3, 61–67. 21160519 10.1038/nchem.891

[263] A. Basagni , F. Sedona , C. A. Pignedoli , M. Cattelan , L. Nicolas , M. Casarin , M. Sambi , J. Am. Chem. Soc. 2015, 137, 1802–1808. 25582946 10.1021/ja510292b

[264] J. Cai , P. Ruffieux , R. Jaafar , M. Bieri , T. Braun , S. Blankenburg , M. Muoth , A. P. Seitsonen , M. Saleh , X. Feng , et al., Nature 2010, 466, 470–473. 20651687 10.1038/nature09211

[265] Z. Chen , H. I. Wang , J. Teyssandier , K. S. Mali , T. Dumslaff , I. Ivanov , W. Zhang , P. Ruffieux , R. Fasel , H. J. Räder , et al., J. Am. Chem. Soc. 2017, 139, 3635–3638. 28248492 10.1021/jacs.7b00776

[266] S. Kawai , S. Saito , S. Osumi , S. Yamaguchi , A. S. Foster , P. Spijker , E. Meyer , Nat. Commun. 2015, 6, 8098. 26302943 10.1038/ncomms9098 PMC4560828

[267] X. Y. Wang , J. I. Urgel , G. B. Barin , K. Eimre , M. Di Giovannantonio , A. Milani , M. Tommasini , C. A. Pignedoli , P. Ruffieux , X. Feng , et al., J. Am. Chem. Soc. 2018, 140, 9104–9107. 29990420 10.1021/jacs.8b06210

[268] C. Bronner , S. Stremlau , M. Gille , F. Brauße , A. Haase , S. Hecht , P. Tegeder , Angew. Chem. Int. Ed. 2013, 52, 4422–4425; 10.1002/anie.201209735 23512734

[269] D. J. Rizzo , G. Veber , T. Cao , C. Bronner , T. Chen , F. Zhao , H. Rodriguez , S. G. Louie , M. F. Crommie , F. R. Fischer , Nature 2018, 560, 204–208. 30089918 10.1038/s41586-018-0376-8

[270] P. Ruffieux , S. Wang , B. Yang , C. Sanchez-Sanchez , J. Liu , T. Dienel , L. Talirz , P. Shinde , C. A. Pignedoli , D. Passerone , et al., Nature 2016, 531, 489–492. 27008967 10.1038/nature17151

[271] M. Di Giovannantonio , J. I. Urgel , U. Beser , A. V. Yakutovich , J. Wilhelm , C. A. Pignedoli , P. Ruffieux , A. Narita , K. Müllen , R. Fasel , J. Am. Chem. Soc. 2018, 140, 3532–3536. 29474072 10.1021/jacs.8b00587

[272] S. Mishra , T. G. Lohr , C. A. Pignedoli , J. Liu , R. Berger , J. I. Urgel , K. Müllen , X. Feng , P. Ruffieux , R. Fasel , ACS Nano 2018, 12, 11917–11927. 30395436 10.1021/acsnano.8b07225

[273] K. Xu , J. I. Urgel , K. Eimre , M. Di Giovannantonio , A. Keerthi , H. Komber , S. Wang , A. Narita , R. Berger , P. Ruffieux , et al., J. Am. Chem. Soc. 2019, 141, 7726–7730. 31046260 10.1021/jacs.9b03554 PMC6557540

[274] S. H. Pun , Y. Wang , M. Chu , C. K. Chan , Y. Li , Z. Liu , Q. Miao , J. Am. Chem. Soc. 2019, 141, 9680–9686. 31132255 10.1021/jacs.9b03910

[275] M. Navakouski , H. Zhylitskaya , P. J. Chmielewski , T. Lis , J. Cybińska , M. Stępień , Angew. Chem. Int. Ed. 2019, 58, 4929–4933; 10.1002/anie.201900175 30714666

[276] J. I. Urgel , M. Di Giovannantonio , Y. Segawa , P. Ruffieux , L. T. Scott , C. A. Pignedoli , K. Itami , R. Fasel , J. Am. Chem. Soc. 2019, 141, 13158–13164. 31340123 10.1021/jacs.9b05501

