# Supplementary material for: Synthetic Applications of Oxidative Aromatic Coupling—From Biphenols to Nanographenes
Source: Angew Chem Int Ed Engl. 2019 Dec 3;59(8):2998–3027. doi: 10.1002/anie.201904934 (PMC7027897; doi:10.1002/anie.201904934)
Supplement: Supplementary file 1 — Supplementary [file ANIE-59-2998-s001.pdf]

Angewandte Chemie
Eine Zeitschrift der Gesellschaft Deutscher Chemiker

Supporting Information

Synthetic Applications of Oxidative Aromatic Coupling—From Biphenols to Nanographenes

Marek Grzybowski, Bartłomiej Sadowski, Holger Butenschön, and Daniel T. Gryko**

anie_201904934_sm_miscellaneous_information.pdf

Author Contributions

M.G. Conceptualization: Equal; Writing - Original Draft: Lead; Writing - Review & Editing: Equal
B.S. Conceptualization: Equal; Writing - Original Draft: Lead; Writing - Review & Editing: Equal
D.G. Conceptualization: Lead; Supervision: Lead; Writing - Original Draft: Equal; Writing - Review & Editing: Lead.
H.B. Conceptualization: Lead; Supervision: Lead; Writing - Original Draft: Equal; Writing - Review & Editing: Lead.

1. Synthesis of small-molecule targets

Intramolecular oxidative coupling of arenes is a convenient method for the synthesis of small-molecule targets such as natural products and drug intermediates, among others. This method is most commonly used for the preparation of 6-membered (benzene) rings, however, in recent years multiple examples of oxidative ring closures of 5-, 7- and 8-membered rings have been reported. The most interesting examples will be discussed in this section.

As demonstrated by Waldvogel and co-workers, $MoCl_5$ and other Mo^{V} reagents are versatile oxidants for both inter- and intramolecular oxidative couplings of arenes.[S1] Recently it was shown that fluorene derivatives **S1**-**S4** can be readily synthesized from the corresponding diarylmethanes *via* Mo^{V}-mediated oxidative closure of 5-membered rings (Figure S1).[S2] In general, electron-rich aromatic moieties are required for this reaction to occur. Using $MoCl_5$ as an oxidant, a series of symmetrically or unsymmetrically substituted spirobifluorenes of type **S1** have been prepared in excellent yields. In addition, the authors obtained 9,9-dimethyl-2,3,6,7-tetramethoxyfluorene (**S2**) and spiro-lactam **S3**. $MoCl_5$ or its analogue with two chlorides substituted with the hexafluoroisopropoxide ligands ($MoCl_3[OCH(CF_3)_2]_2$) have also been used in the synthesis of a library of 9-monosubstituted, unsymmetrical fluorenes of the type **S4** (Figure S1).[S3]

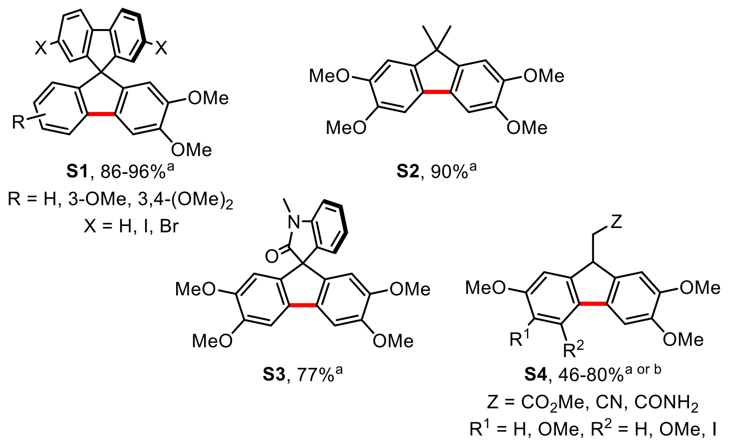

Figure S1. Cyclization conditions: a) $MoCl_5$ (2.2-3.9 eq.), CH_2Cl_2, RT; b) $MoCl_3[OCH(CF_3)_2]_2$ (3 eq.), CH_2Cl_2, RT.

The HFIP-modified oxidant generally leads to higher yields of the corresponding fluorenes with reduced amounts of chlorinated side-products, which had been demonstrated earlier on a broader scope of inter- and intramolecular oxidative couplings.[S4] A very good example is the oxidation of benzothienyl-substituted fluorene **S5**, which with $MoCl_5$ proceeds with the formation of chlorinated spiro-bifluorene analogue **S6a** in high yield, whereas $MoCl_3[OCH(CF_3)_2]_2$ efficiently yields the non-chlorinated product **S6b** (Scheme S1).[S5] Similar to fluorenes, carbazoles can also be synthesized from the corresponding di- or triarylamines by Mo^V-mediated oxidative closure of 5-membered ring.[S6]

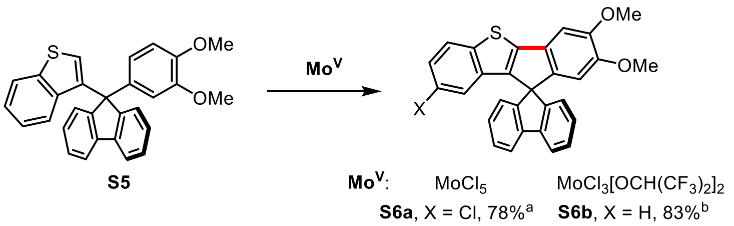

Scheme S1. Reaction conditions: a) $MoCl_5$ (3 eq.), CH_2Cl_2, RT; b) $MoCl_3[OCH(CF_3)_2]_2$ (2 eq.), CH_2Cl_2, RT.

Figure S2 depicts some of the small-molecular targets which can be synthesized by oxidative closure of 6-membered rings. As reported by Pelkey and co-workers, PIFA/$BF_3 \cdot Et_2O$ is a powerful system for the synthesis of phenanthrene derivatives of the type **S7**,[S7] as well as its heterocyclic analogues **S8** and **S9** containing indole moieties.[S8] These compounds are structurally related to the natural product staurosporine, an inhibitor of protein kinase C, and some of them show enhanced biological activities.[S7,S8]

Phenanthrene derivatives such as **S10a**-**c** are considered as synthetic intermediates for important anti-cancer alkaloids from the phenanthro-quinolizidine and -indolizidine families, i.e. tylophorine and cryptopleurine.[S9] The polymethoxy-substituted phenanthrene skeleton can be readily prepared in high yields by cyclization from the corresponding stilbene derivatives using various oxidative conditions: $MoCl_5$ or $MoCl_5$/$TiCl_4$,[S9] CAN,[S10] or dehydrogenation with 5% Pd on Al_2O_3 in an atmosphere of oxygen (**S10a-c**, Figure S2).[S11] The presence of $TiCl_4$ in the $MoCl_5$-mediated oxidation greatly improves the yield of phenanthrene **S10a** from 80% to 98%.[S9] Higher yields are attributed to the HCl-scavenging properties of $TiCl_4$, which minimizes unwanted chlorination.[S12] Dehydrogenation with Pd/Al_2O_3 under oxygen proved to be an excellent method for the synthesis of triphenylenes, such as **S11a**-**g**.[S11] A similar range of triphenylene derivatives can also be obtained through indirect anodic oxidation in the presence of 5 mol% of DDQ as a redox mediator.[S13]

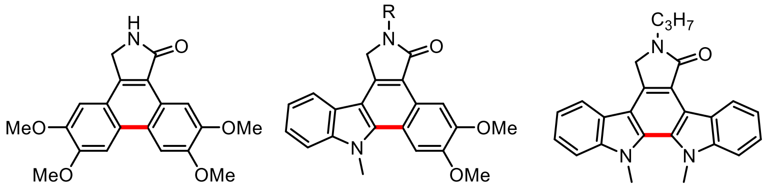

S7, 96%[a]

S8a, R = Pr, 55%[a]
S8b, R = H, 83%[a]

S9, 69%[a]

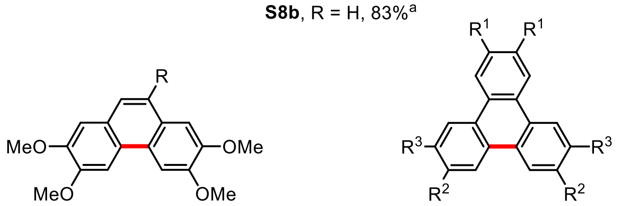

S10a, R = CO_2Et, 80%[b] / 98%[c]
S10b, R = CO_2Me, 87%[d]
S10c, R = H, 85%[d] / 54%[e]
R = CN, CHO, $CONR_2$, 78-89%[d]

S11a, $R^1 = R^2 = R^3$ = H, 96%[e]
S11b, $R^1 = R^2$ = H, R^3 = Me, 52%[e]
S11c, $R^1 = R^2$ = H, R^3 = Ph, >99%[e]
S11d, $R^1 = R^2$ = H, R^3 = OMe, 83%[e]
S11e, R^1 = H, $R^2 = R^3$ = OMe, >99%[e]
S11f, $R^1 = R^2$ = OMe, R^3 = H, 43%[e]
S11g, $R^1 = R^2 = R^3$ = OMe, >99%[e]

Figure S2. Selected examples of oxidative 6-membered ring closures. Reaction conditions: a) PIFA (1.1 eq.), $BF_3{\cdot}Et_2O$ (1.2 eq.), CH_2Cl_2, −40 °C; b) $MoCl_5$ (2.2 eq.), CH_2Cl_2, RT; c) $MoCl_5$ (2.2 eq.), $TiCl_4$ (2.3 eq.), CH_2Cl_2, 0 °C; d) CAN (2 eq.), $NaHCO_3$ (4 eq.), CH_3CN, RT; e) 5% Pd/Al_2O_3 (5 mol%), O_2 (1 atm), TfOH or MsOH, TFE or HFIP, RT.

Similar cyclizations involving two thienyl groups in the formation of 6-membered rings were performed using $FeCl_3$, $MoCl_5$ or DDQ as oxidants in the syntheses of various sulfur-doped PAHs.[S5,S14–S20]

DDQ[S21] or MnO_2[S22] in the presence of $BF_3{\cdot}Et_2O$ enable the efficient oxidative closure of 7- and 8-membered rings leading to compounds **S12a-d** and **S13a-d**, respectively, when precursors bearing electron-rich aryl substituents are used as the starting materials (Figure S3).

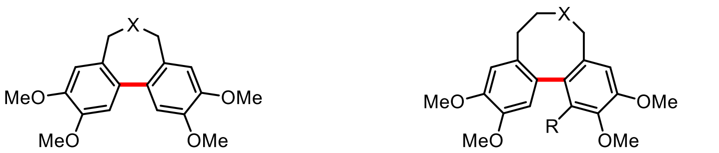

S12a, X = $NCOCF_3$, 95%[a] / 98%[b]
S12b, X = O, 56%[a] / 83%[b]
S12c, X = SO, 65%[a] / 78%[b]
S12d, X = CH_2, 98%[b]

S13a, X = $NCOCF_3$, R = H, 96%[a]
S13b, X = $NCOCF_3$, R = OMe, 90%[a]
S13c, X = CH_2, R = H, 92%[a] / 80%[b]
S13d, X = CH_2, R = OMe, 90%[a] / 80%[b]

Figure S3. Oxidative closure of 7- and 8-membered rings. Reaction conditions: a) DDQ (1 eq.), $BF_3{\cdot}Et_2O$ (12 eq.), CH_2Cl_2, RT; b) MnO_2 (6 eq.), $BF_3{\cdot}Et_2O$ (3 eq.), CH_2Cl_2, RT.

An interesting method for the oxidative construction of 7-membered rings has been discovered by Du, Zhao and co-workers (Scheme S2).[S23] Upon treatment with PIDA in boiling acetonitrile, benzanilides **S14**, bearing an acetylated amino group at the *ortho*-position relative to the benzanilide carbonyl, underwent oxidative cyclization accompanied by a ring-expanding rearrangement to give biaryl-bridged, 7-membered ring-based ureas **S15** in moderate to good yields. Some mechanistic insights into this process were provided by the fact that the analogous transformation proceeds for hydroxybenzanilides **S16** with PIFA as an oxidant. In this case, however, the reaction is a two-step process and addition of $BF_3{\cdot}Et_2O$ is necessary to convert the initially forming spirocyclic intermediate **S17** into the cyclic carbamates **S18**. The authors suggested that the formation of ureas **S15** also involves a 5-membered-ring intermediate.

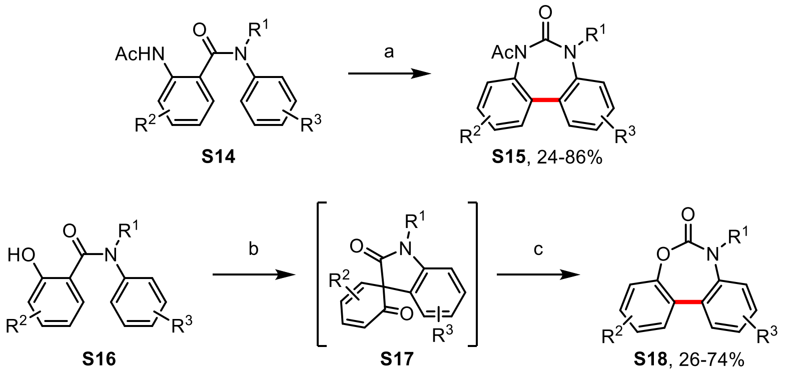

R^1 = Alkyl, Aryl; R^2 and R^3 = H, F, Cl, Br, OMe, Alkyl

Scheme S2. Oxidative coupling with a rearrangement to a 7-membered ring. Reaction conditions: a) PIDA (3 eq.), CH_3CN, reflux; b) PIFA (1.3 eq.), CH_2Cl_2, -35 °C or 0 °C; c) $BF_3\cdot Et_2O$ (0.3 eq.), RT.

References

[S1] S. R. Waldvogel, S. Trosien, *Chem. Commun.* **2012**, *48*, 9109–9119.

[S2] S. Trosien, D. Schollmeyer, S. R. Waldvogel, *Synthesis* **2013**, *45*, 1160–1164.

[S3] P. Franzmann, S. Trosien, M. Schubert, S. R. Waldvogel, *Org. Lett.* **2016**, *18*, 1182–1185.

[S4] M. Schubert, J. Leppin, K. Wehming, D. Schollmeyer, K. Heinze, S. R. Waldvogel, *Angew. Chem. Int. Ed.* **2014**, *53*, 2494–2497.

[S5] M. Schubert, S. Trosien, L. Schulz, C. Brscheid, D. Schollmeyer, S. R. Waldvogel, *Eur. J. Org. Chem.* **2014**, *2014*, 7091–7094.

[S6] S. Trosien, P. Böttger, S. R. Waldvogel, *Org. Lett.* **2014**, *16*, 402–405.

[S7] A. A. Van Loon, M. K. Holton, C. R. Downey, T. M. White, C. E. Rolph, S. R. Bruening, G. Li, K. M. Delaney, S. J. Pelkey, E. T. Pelkey, *J. Org. Chem.* **2014**, *79*, 8049–8058.

[S8] N. J. Truax, F. Banales Mejia, D. O. Kwansare, M. M. Lafferty, M. H. Kean, E. T. Pelkey, *J. Org. Chem.* **2016**, *81*, 6808–6815.

[S9] K. Wehming, M. Schubert, G. Schnakenburg, S. R. Waldvogel, *Chem. Eur. J.* **2014**, *20*, 12463–12469.

[S10] V. Gupta, V. U. B. Rao, T. Das, K. Vanka, R. P. Singh, *J. Org. Chem.* **2016**, *81*, 5663–5669.

[S11] S. Fujimoto, K. Matsumoto, M. Shindo, *Adv. Synth. Catal.* **2016**, *358*, 3057–3061.

[S12] B. Kramer, R. Fröhlich, S. R. Waldvogel, *Eur. J. Org. Chem.* **2003**, 3549–3554.

[S13] P. Röse, S. Emge, C. A. König, G. Hilt, *Adv. Synth. Catal.* **2017**, *359*, 1359–1372.

[S14] D. Waghray, C. De Vet, K. Karypidou, W. Dehaen, *J. Org. Chem.* **2013**, *78*, 11147–11154.

[S15] Z. Li, J. Zhang, K. Zhang, W. Zhang, L. Guo, J. Huang, G. Yu, M. S. Wong, *J. Mater. Chem. C* **2015**, *3*, 8024–8029.

[S16] R. Q. Lu, Y. N. Zhou, X. Y. Yan, K. Shi, Y. Q. Zheng, M. Luo, X. C. Wang, J. Pei, H. Xia, L. Zoppi, et al., *Chem. Commun.* **2015**, *51*, 1681–1684.

[S17] W. Zhang, X. Sun, P. Xia, J. Huang, G. Yu, M. S. Wong, Y. Liu, D. Zhu, *Org. Lett.* **2012**, *14*, 4382–4385.

[S18] C. Quinton, M. Suzuki, Y. Kaneshige, Y. Tatenaka, C. Katagiri, Y. Yamaguchi, D. Kuzuhara, N. Aratani, K. Nakayama, H. Yamada, *J. Mater. Chem. C* **2015**, *3*, 5995–6005.

[S19] Q. Ye, Z. Zhang, Z. M. Png, W. T. Neo, T. Lin, H. Zeng, H. Xu, J. Xu, *J. Org. Chem.* **2016**, *81*, 9219–9226.

[S20] L. Zöphel, V. Enkelmann, R. Rieger, K. Müllen, *Org. Lett.* **2011**, *13*, 4506–4509.

[S21] S. Sun, J. Yang, F. Li, Z. Lv, W. Li, H. Lou, L. Liu, *Tetrahedron Lett.* **2014**, *55*, 6899–6902.

[S22] J. Yang, S. Sun, Z. Zeng, H. Zheng, W. Li, H. Lou, L. Liu, *Org. Biomol. Chem.* **2014**, *12*, 7774–7779.

[S23] S. Shang, D. Zhang-Negrerie, Y. Du, K. Zhao, *Angew. Chem. Int. Ed.* **2014**, *53*, 6216–6219.
